# Re-Evaluation of the Taxonomy of *Talaromyces minioluteus*

**DOI:** 10.3390/jof7110993

**Published:** 2021-11-20

**Authors:** Ioanna Pyrri, Cobus M. Visagie, Piera Soccio, Jos Houbraken

**Affiliations:** 1 Section of Ecology and Systematics, Department of Biology, School of Science, National and Kapodistrian University of Athens, Panepistimioupoli, GR-157 84 Athens, Greece; 2 Department of Biochemistry, Genetics and Microbiology, Forestry and Agricultural Biotechnology Institute (FABI), University of Pretoria, Pretoria 0028, South Africa; cobus.visagie@fabi.up.ac.za; 3 Respiratory Diseases, Department of Medical and Surgical Sciences, University of Foggia, “Policlinico Riuniti” of Foggia, 71122 Foggia, Italy; pierasoccio88@gmail.com; 4 Westerdijk Fungal Biodiversity Institute, Uppsalalaan 8, 3584 CT Utrecht, The Netherlands

**Keywords:** identification, phylogeny, new species, *Eurotiales*

## Abstract

*Talaromyces minioluteus* belongs to the section *Trachyspermi*, has a worldwide distribution and has been found on various substrates, especially on various (stored) food commodities and indoor environments. This species is phenotypically and phylogenetically closely related to *T. chongqingensis* and *T. minnesotensis*. The phylogenetic and morphological analyses of 37 strains previously identified as *T. chongqingensis*, *T. minnesotensis* and *T. minioluteus* revealed that this clade incudes eight species: the accepted species *T. chongqingensis*, *T. minnesotensis* and *T. minioluteus*, the newly proposed species *T. calidominioluteus*, *T. africanus* and *T. germanicus*, and the new combinations *T. gaditanus* (basionym *Penicillium gaditanum*) and *T. samsonii* (basionym *Penicillium samsonii*). In this study, we give insight of the phylogenetic relationships and provide detailed descriptions of the species belonging to this clade. Macromorphological features, especially colony growth rates, texture and conidial colors on agar media, are important characters for phenotypic differentiation between species.

## 1. Introduction

*Talaromyces minioluteus* belongs to the section of *Trachyspermi* and is phylogenetically related to *T. chongqingensis*, *T. minnesotensis* and *T. udagawae.* It has a worldwide distribution and is previously isolated from various substrates, including soil, indoor environments (e.g., air, dust) and various foods, and has been reported as a postharvest pathogen of quince, tomato and citrus fruits [1]. Various mycotoxins (e.g., rubratoxin, rugulosin, secalonic acid, skyrin) and other extrolites (e.g., minioluteic acid, spiculisporic acid, mitorubrinic acid, mitorubrin, mitorubrinol, mitorubrinol-acetate) can be produced by this species [2,3,4].

In the past, based on a morphological approach, Pitt [5] introduced an infrageneric classification system in *Penicillium* with four subgenera. *Penicillium* subgenus *Biverticillium* species (most currently classified in *Talaromyces*) were characterized by its symmetrically, biverticillate conidiophores with acerose phialides and having slow growth on 25% glycerol nitrate agar. Early molecular analysis showed that these species were phylogenetically distinct from species classified in the other three subgenera [6,7]. *Penicillium* was polyphyletic with the inclusion of subgenus *Biverticillium* and it was thus suggested to be excluded from *Penicillium* [7]. Later multigene analyses confirmed this result and demonstrated that the majority of *Talaromyces* and subgenus *Biverticillium* species cluster together, forming a monophyletic clade within the *Trichocomaceae* [8,9]. Based on these results and in the light of the single name nomenclature concept, *Talaromyces* was redefined to also include the majority of species previously classified in subgenus *Biverticillium* [10] and for example, *Penicillium minioluteum* was given the new combination *Talaromyces minioluteus*.

*Talaromyces minioluteus* was originally described by Dierckx [11] as forming white colonies with grey-green spots and having a yellow-red reverse that turns brown-grey in time. Raper and Thom [12] synonymized *P. minioluteum* with *P. funiculosum* (currently *T. funiculosus*) based on examination of Biourge’s *P. minioluteum* strain. In contrast to Raper and Thom [12], Pitt [5] accepted *P. minioluteum* and this was confirmed in the taxonomic study of van Reenen-Hoekstra et al. [3]. In contrast to *P. funiculosum*, *P. minioluteum* produces more restricted colonies with striking yellow mycelium and does not, or slowly, grows at 37 °C. Furthermore, *T. minioluteus* strains can produce red soluble pigments, though not consistently [4]. Pitt [5] also neotypified *P. minioluteum* with Biourge’s strain (IMI 89377ii). The ex-neotype strains of *P. minioluteum* FRR 1714 (=CBS 196.88) and CBS 642.68 differ morphologically causing confusion in the taxonomy of this species. The latter strain fully fits Dierckx’s original description and is therefore considered correct [3]; CBS 196.88 was shown to be *T. ruber* [13].

*Penicillium gaditanum*, *Penicillium samsonii* and *Penicillium purpurogenum* var. *rubrisclerotium* are related to *T. minioluteus* [3,4]. *Penicillium gaditanum* was originally described in the *P. funiculosum* series [12,14], while *P. samsonii* was described in series *Islandica* (sect. *Simplicium*) [5,15]. Based on morphology and extrolite data, both species were synonymized with *P. minioluteum* [3]. *Penicillium gaditanum* was not included in the monographic treatment of *Talaromyces* [4] and van Reenen-Hoekstra et al. [3] was followed, while the synonymy of *P. samsonii* with *T. minioluteus* was confirmed. The taxonomic position of *P. purpurogenum* var. *rubrisclerotium* is unclear. Yilmaz et al. [13] noted that many strains previously identified as *P. purpurogenum* var. *rubrisclerotium* resolved in a clade with *T. amestolkiae*, but CBS 270.35, the ex-type strain of this species resolved in a distinct clade closely related to *T. minioluteus*. *Talaromyces minnesotensis* and *T. chongqingensis* are two recently described species related to *T. minioluteus* [16,17].

Based on the analysis of internal transcribed spacer rDNA region (ITS) and β-tubulin (*BenA*) sequences, Visagie et al. [18] suggested that *T. minioluteus* represents a species complex. The objective of our study is to resolve the taxonomic status of strains previously assigned to *T. minioluteus* using a polyphasic approach that includes macro- and micro-morphological data combined with the phylogenetic analysis of *BenA*, calmodulin (*CaM*), ITS and RNA polymerase II second largest subunit (*RPB2*) gene sequences. We show that the *T. minioluteus*-clade includes eight species (three accepted species, three new species and two new combinations) and detailed descriptions of these species and the phylogenetically related *T. udagawae* are provided here.

## 2. Materials and Methods

### 2.1. Strains

The studied strains were obtained from the CBS culture collection (CBS) and the internal working collection of the Food and Indoor Mycology department (DTO), both housed at the Westerdijk Fungal Biodiversity Institute, Utrecht, The Netherlands. The strain selection was based on a homology search in these collections using ITS, *BenA* or *CaM* sequences. Strains phylogenetically related to *T. minioluteus* were selected for a more detailed analysis. Τhe strains originate from different sources and countries and more details are given in Table 1.

### 2.2. DNA Isolation, Amplification and Sequence Analysis

Total genomic DNA was extracted from colonies grown on malt extract agar (MEA) for 4 d with the DNeasy^®^ UltraClean^®^ Microbial Kit (Qiagen, Germany) according to manufacturer instructions. The genetic markers *BenA*, *CaM*, ITS and *RPB2* were amplified using the primer pairs Bt2a/Bt2b [19], CMD5/CMD6 [20], V9G/LS266 [21,22] and RPB2-F1/RPB2-7CR_1 [9] (alternatively RPB2-5F_Eur/RPB2-7CR_Eur [23]), respectively. PCR reactions were performed in a 13 μL total volume containing 0.63 μL dimethylsulfoxide (DMSO, 5% *w/w*), in which 1 μL of template DNA was mixed with 0.98 μL dNTPs (1 mM), 0.25 μL of each primer (10 μM) and 0.05 μL of BioTaq DNA polymerase (5 U μL). The MgCl_2_ concentration was 1.46 mM. The targeted loci were amplified with a cycle program comprising an initial denaturation step at 95 °C for 5 min, followed by 35 cycles of 30 s at 95 °C (denaturation), 40 s at 55 °C (primer annealing) for *benA*, *CaM* and ITS and 48 and 52 °C for *RPB2*, and 2 min at 72 °C (primer extension) and ending with a final extension of 7 min at 72 °C. Automated sequencing of both strands of the PCR amplicons was performed on a 3730xl DNA Sequencer (Thermo Fisher Scientific, Headquarters: Waltham, MA, USA) using the BigDye Terminator chemistry. The primers in the sequencing reactions were the same as those used for PCR amplifications. Consensus sequences for each locus were assembled using SeqMan Pro v.15 (DNASTAR, Madison, WI, USA). Novel sequences generated in this study were deposited in the NCBI nucleotide database (GenBank) under accession numbers depicted in Table 1.

Single gene alignments were generated in MAFFT v. 7 [24] and subsequently trimmed at both ends in MEGA7 [25]. Phylogenetic analysis was performed based on the individual *BenA*, *CaM*, ITS and *RPB2* sequence datasets as well as on the combined *BenA*, *CaM*, ITS and *RPB2* dataset. The individual loci in the combined dataset were analysed as separate partitions. Phylogenetic trees were constructed based on Maximum Likelihood (ML) using RAxML-HPC2 on XSEDE 8.2.10 [26] through the CIPRES Science Gateway v.3.3 [27] and Bayesian Inference (BI) with MrBayes v. 3.2 [28]. The substitution model used in both analyses was GTR with gamma distribution and invariable sites. For ML bootstrap supports were estimated by 1000 replicates. Bayesian analysis was run with four MCMC chains for 10,000,000 generations and stopped when the average standard deviation of split frequencies was <0.01. Burn in was set to 25% after which the likelihood values were stationary. Random trees were sampled every 100 generations. The resulting trees were visualized with TreeView v1.6.6 and edited in Adobe Illustrator CS5. Bayesian inference (BI) posterior probabilities (pp) values and bootstrap (bs) values are labelled on the nodes. Values less than 0.95 pp and 70% bs are not shown.

In order to get insight in the distribution of the investigated species, *BenA* and ITS sequences deposited in GenBank and belonging to the *T. minioluteus*-clade were re-analyzed. These sequences were merged in our dataset, aligned and a phylogram was constructed according to the methods described above.

### 2.3. Morphology

The selected strains were three-point inoculated on the agar media Czapek yeast autolysate agar (CYA), MEA (Oxoid), yeast extract sucrose agar (YES), creatine sucrose agar (CREA), CYA supplemented with 5% NaCl (CYAS), dichloran 18% glycerol agar (DG18) and oatmeal agar (OA). Agar media formulations are as described by Samson et al. (2014) [29]. Plates were incubated for 7 d at 25 °C in the dark. Additional CYA plates were incubated at 30 and 37 °C. Colony growth rate, colony texture, degree of sporulation, obverse and reverse colony colors, production of soluble pigments and exudates on all media were determined and recorded after 7 d of incubation. Color names and codes follow Kornerup and Wanscher [30]. Colonies were photographed with a Nikon D3200 camera. Microscopic observations were made in mounts prepared from colonies grown on MEA after the application of 96% ethanol to remove excess conidia and using lactic acid (60%) as mounting medium. Observations and images were made with a Nikon SMZ25 stereo-microscope and a Zeiss AxioImager.A2 with Differential Interference Contrast (DIC) microscopy, both equipped with Nikon DS-Ri2 cameras.

## 3. Results

### 3.1. Phylogeny

**Figure 1:**
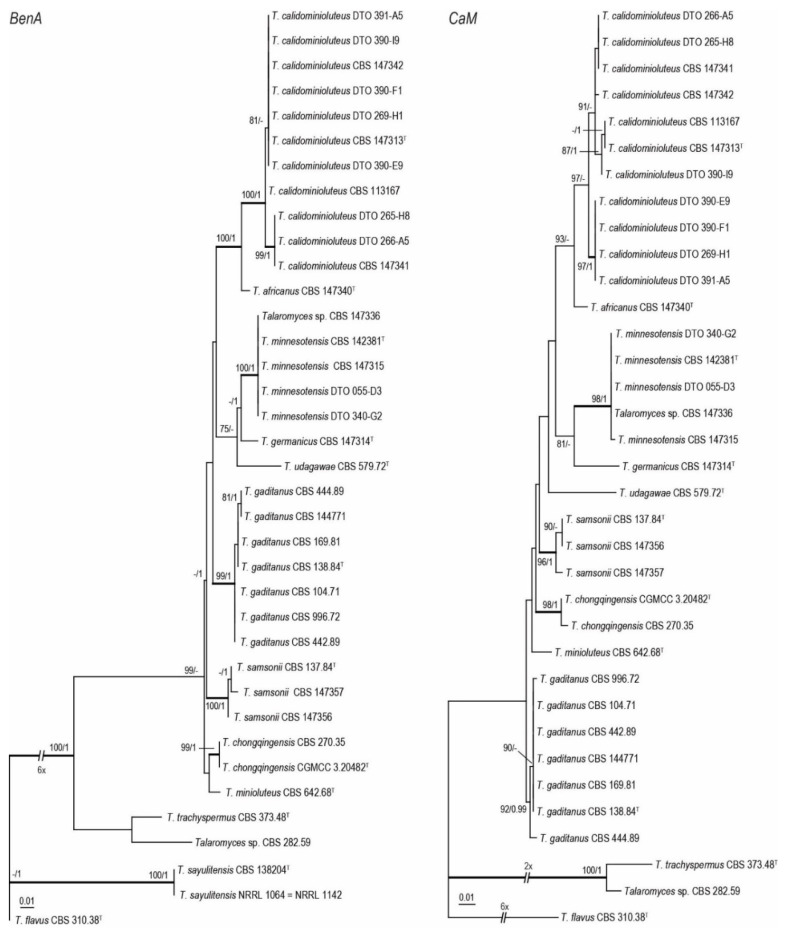
Maximum Likelihood (ML) tree based on *BenA* and *CaM* gene sequences depicting the relationships of *T. minioluteus* and related species. The BI posterior probabilities and ML bootstrap percentages are presented at the nodes (BS/PP) and the fully supported branches are thickened. Values less than 70% bs (ML) or less than 0.95 pp (Bayesian analysis) are indicated with a hyphen or not shown. The *BenA* phylogram is rooted with *Talaromyces flavus* (CBS 310.38^T^), *T. sayulitensis* (CBS 138204^T^, NRRL 1142 [=NRRL 1064]), *T. trachyspermus* (CBS 373.48^T^) and *Talaromyces* sp. (CBS 282.59); the *CaM* phylogram with *Talaromyces flavus* (CBS 310.38^T^), *T. trachyspermus* (CBS 373.48^T^) and *Talaromyces* sp. (CBS 282.59). ^T^ = ex-type.

**Figure 2:**
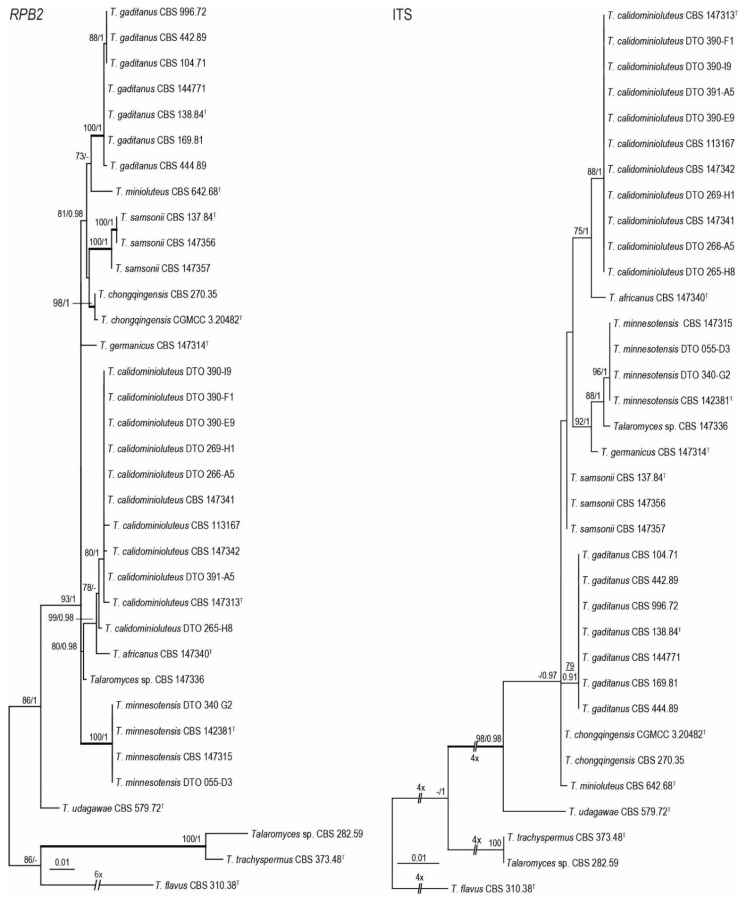
Maximum Likelihood (ML) tree based on ITS and *RPB2* sequences depicting the relationship of *T. minioluteus* and related species. The BI posterior probabilities and ML bootstrap percentages are presented at the nodes (BS/PP) and the fully supported branches are thickened. Values less than 70% bs (ML) or less than 0.95 pp (Bayesian analysis) are indicated with a hyphen or not shown. The phylograms are rooted with *T. flavus* (CBS 310.38^T^), *T. trachyspermus* (CBS 373.48^T^) and *Talaromyces* sp. (CBS 282.59). ^T^ = ex-type.

**Figure 3:**
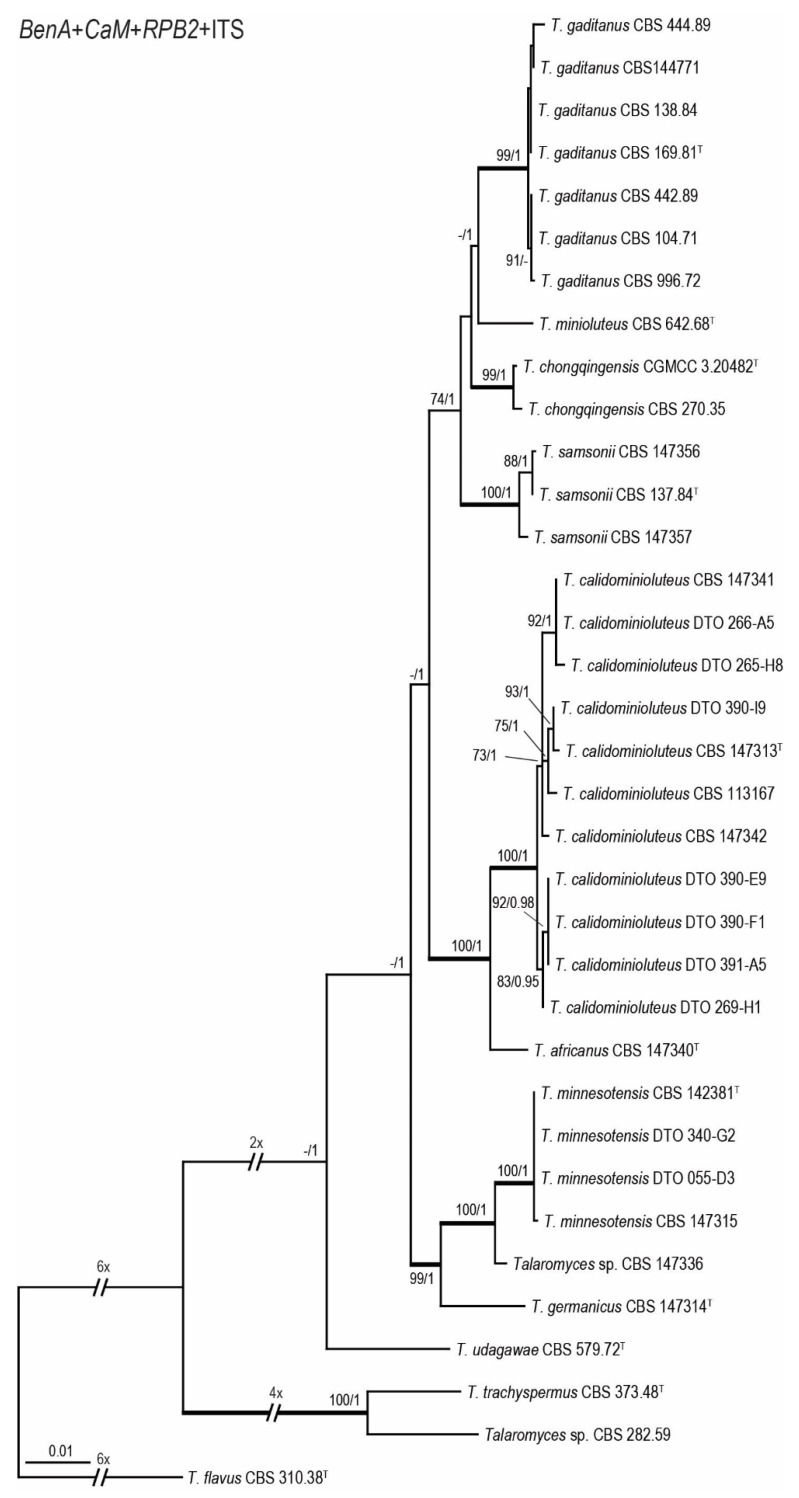
Maximum Likelihood (ML) tree inferred from the combined *BenA*, *CaM*, ITS and *RPB2* sequence dataset depicting the relationship of *T. minioluteus* and phylogenetically related species. The BI posterior probabilities and ML bootstrap percentages are presented at the nodes (BS/PP) and the fully supported branches are thickened. Values less than 70% bs (ML) or less than 0.95 pp (Bayesian analysis) are indicated with a hyphen or not shown. The phylogram is rooted with *T. flavus* (CBS 310.38^T^), *T. trachyspermus* (CBS 373.48^T^) and *Talaromyces* sp. (CBS 282.59). ^T^ = ex-type.

Phylogenetic relationships within the *T. minioluteus*-clade were inferred using single gene sequence datasets of the four studied genetic markers (Figure 1 and Figure 2), as well as the combined dataset containing *BenA*, *CaM*, ITS and *RPB2* gene sequences (Figure 3). In total, 37 strains were used for the *BenA* analysis and 35 in the other datasets. The total lengths of the aligned datasets were 491, 616, 621 and 954 base pairs for *BenA*, *CaM*, *ITS* and *RPB2*, respectively.

The phylogenies inferred on the *BenA*, *CaM*, ITS and *RPB2* datasets were congruent. These data show that *T. minioluteus* comprise a clade with phylogenetically related, but distinct, species. Phylogenetic analysis revealed nine lineages (considered here as nine species), including the phenotypically distinct species *T. udagawae*. Four lineages are represented by the accepted species *T. chongqingensis*, *T. minioluteus*, *T. minnesotensis* and *T. udagawae*. The ex-type strains of *P. gaditanum* (CBS 169.81) and *P. samsonii* (CBS 137.84) are located in two distinct lineages and are combined in *Talaromyces* (as *T. gaditanus* and *T. samsonii*, see taxonomy). The new species *T. africanus*, *T. calidominioluteus* and *T. germanicus* are proposed for the three remaining lineages. The multi-gene phylogeny resolved the studied strains into four main clades. The segregation into these four clades is strongly supported by Bayesian Inference, while statistical support is lacking in the ML analysis. The most species rich clade includes *T. minioluteus*, *T. gaditanus*, *T. chongqingensis* and *T. samsonii* and is sister to the clade that includes the new species *T. calidominioluteus* and *T. africanus*. The sister to these is a clade that contains *T. minnesotensis* and *T. germanicus*. The phenotypically distinct species *T. udagawae* takes a basal position to the other three clades.

### 3.2. Morphology

**Figure 4:**
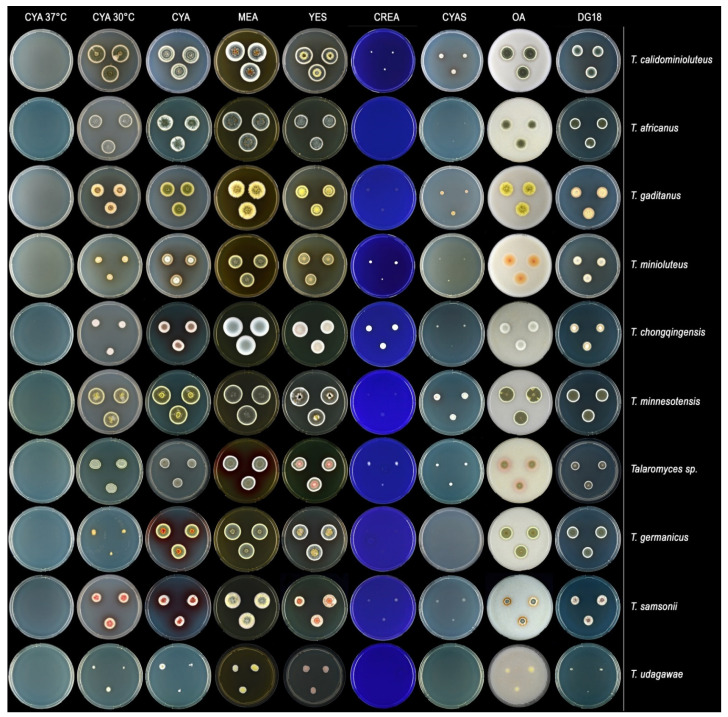
Left to right: 7 d old colonies on CYA 37 °C, CYA 30 °C, CYA, MEA, YES, CREA, CYAS, OA, DG18; from top to bottom: *T. calidominioluteus* (DTO 390-E9), *T. africanus* (CBS 147340), *T. gaditanus* (CBS 169.81), *T. minioluteus* (CBS 642.68), *T. chongqingensis* (CBS 147316), *T. minnesotensis* (CBS 141838), *Talaromyces* sp. (CBS 147336), *T. germanicus* (CBS 147314), *T. samsonii* (CBS 137.84), *T. udagawae* (CBS 579.72).

Species from the *T. minioluteus*-clade can be segregated into three groups according to their growth rate on CYA at 30 °C. *Talaromyces calidominioluteus* and *T. africanus* grow faster than the others species (18–29 mm), *T. minioluteus*, *T. samsonii* and *T. gaditanus* grow moderately fast (9–16 mm) whereas *T. germanicus*, *T. chongqingensis* and *T. udagawae* grow the slowest (5–8 mm) (Table 2). The mycelium and conidial color are also informative characters to distinguish species. *Talaromyces minioluteus*, *T. samsonii*, *T. gaditanus*, *T. germanicus* and *T. chongqingensis* produce yellow mycelium and conidia in green shades while *T. calidominioluteus*, *T. africanus* and *T. minnesotensis* have white mycelium and bluish green conidia. Furthermore, the colony texture on MEA is informative, for example *T. africanus* and *T. germanicus* produce velvety colonies while *T. gaditanus* and *T. samsonii* colonies have a floccose texture (Table 2, Figure 4). All species are well differentiated macro-morphologically, especially on CYA, MEA and OA (Figure 4), and generally produce reddish exudates or soluble pigments. The micromorphology of this group of species is highly similar and all produce symmetrically biverticillate conidiophores with (broadly) ellipsoidal or fusiform, smooth-walled conidia. The exception is *T. africanus* that produces subglobose, finely roughened conidia. *Talaromyces udagawae* is phenotypically distinct and the only species that produces a sexual morph.

### 3.3. Taxonomy

Three new species and two new combinations are introduced in this study. All species were compared with their morphologically and phylogenetically closest relatives. Diagnostic characters are summarized in Table 2 and an overview of growth rates on various diagnostic media is shown in Figure 4.

**Figure 5:**
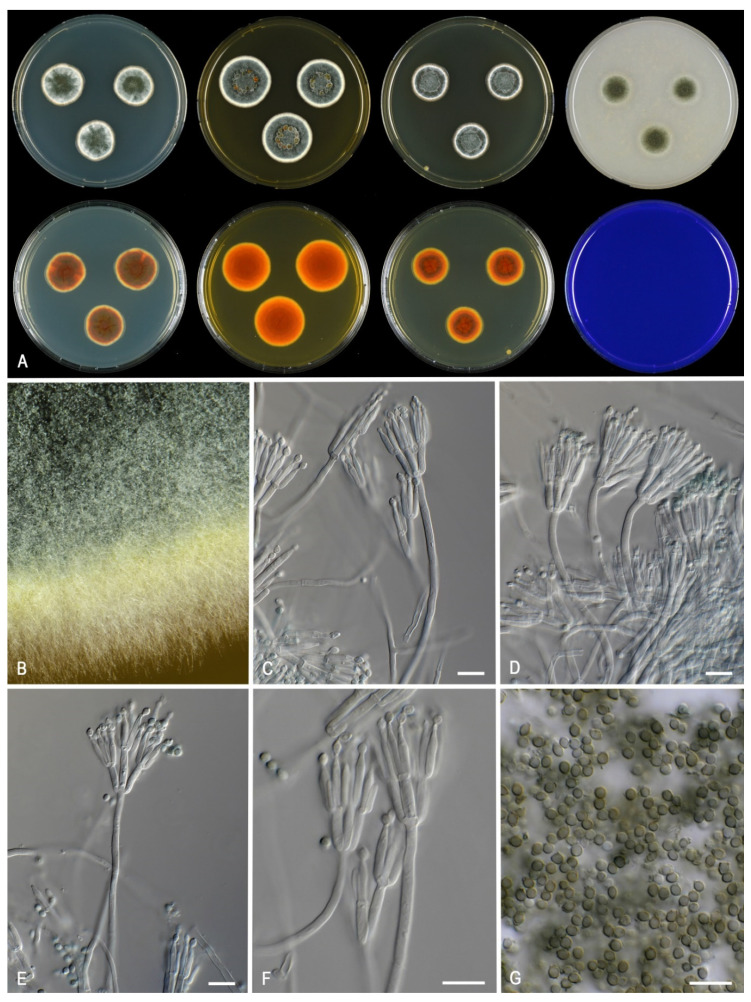
Morphological characters of *Talaromyces africanus* (CBS 147340). (**A**). Colonies, from left to right, after 7 d at 25 °C (top row) CYA, MEA, YES, OA; (bottom row) CYA reverse, MEA reverse, YES reverse, CREA. (**B**). Detail of colony on MEA. (**C**–**F**). Conidiophores and conidia. (**G**). Conidia. Scale bars = 10 µm.

***Talaromyces africanus*** Houbraken, Pyrri and Visagie, *sp. nov.* MycoBank MB 841228. Figure 5.

*Etymology*: Referring to Africa, the continent where the type was isolated.

*Diagnosis*: Colony diameter on CYA incubated at 30 °C 18–20 mm, conidia subglobose, finely roughened.

*Typus*: South Africa, house dust, February 2012, collected and isolated by C.M. Visagie (holotype CBS H-24874, culture ex-type CBS 147340 = DTO 179-C5 = KAS 3859).

ITS barcode: OK339610 (alternative markers: *BenA* = OK338782; *CaM* = OK338808; *RPB2* = OK338833).

*Colony diam (7 d, in mm)*: CREA no growth; CYA 21–23; CYA 30 °C 18–20; CYA 37 °C no growth; CYAS 2–3; DG18 15–17; MEA 26–28; OA 18–20; YES 20–22.

*Colony characters*: *CYA* 25 °C, 7 *d*: Colonies low, radially sulcate with concentric rings, margin entire, plane, moderate; sporulation dense; conidia *en masse* greyish turquoise (24E6); mycelium white; colony texture velvety to weakly floccose; exudates present clear; soluble pigments absent; reverse reddish brown (8E6). *MEA* 25 °C, 7 *d*: Colonies with a few sulcates near the center, low, margin plane, low, wide regular; sporulation dense; conidia *en masse* greyish turquoise (24D5–E5); mycelium white; colony texture velvety to weakly floccose; exudates abundant, yellow; soluble pigments absent; reverse reddish orange to copper red (C8). *YES* 25 °C, 7 *d*: Colonies radially sulcate, raised at the center, margin plane, low, narrow, regular; sporulation dense; conidia *en masse* dull green to greyish green (25E4–5); mycelium white; exudates absent; soluble pigments absent; reverse dark brown (7F4) at the center, copper red (7C8). *DG18* 25 °C, 7 *d*: Colonies slightly radially sulcate at the center, low, margin plane, low, moderate, regular; sporulation strong; conidia *en masse* dark green (25F7–8); mycelium white; colony texture velvety; exudates absent; soluble pigments absent; reverse brownish orange (6C8). *OA* 25 °C, 7 *d*: Colonies plane, low, margins entire, plane, narrow; sporulation moderate to dense; conidia *en masse* dull green to greyish green (27E4–5); mycelium white; colony texture velvety to granulose; exudates clear droplets; soluble pigments absent; *CREA* 25 °C, 7 *d*: no growth.

*Micromorphology:* Sclerotia and ascomata not observed. Conidiophores biverticillate; Stipes smooth-walled, non-vesiculate, 80–200 × 2–3 µm; Metulae adpressed, 3–6 per stipe 10–13.5(–15) × 2.5–3.5 µm; Phialides acerose, 3–8 per metula, 10–14 × 2–3 µm; Conidia in long, distorted chains, finely roughened, occasionally smooth, subglobose, with distinct connectives on both sides, 2.5–3.5 × 2–2.5 µm.

*Notes*: *Talaromyces africanus* is phylogenetically closely related to *T. calidominioluteus* (Figure 3). The new species can be differentiated by more restricted growth on CYA at 25 °C (21–23 vs. 20–30 mm) and 30 °C (18–20 vs. (20–)25–29 mm). The reverse color on CYA is reddish brown whereas *T. calidominioluteus* strains produce greyish orange reverses. *Talaromyces africanus* produces finely roughened, subglobose conidia, a unique feature in the *T. minioluteus*-clade.

**Figure 6:**
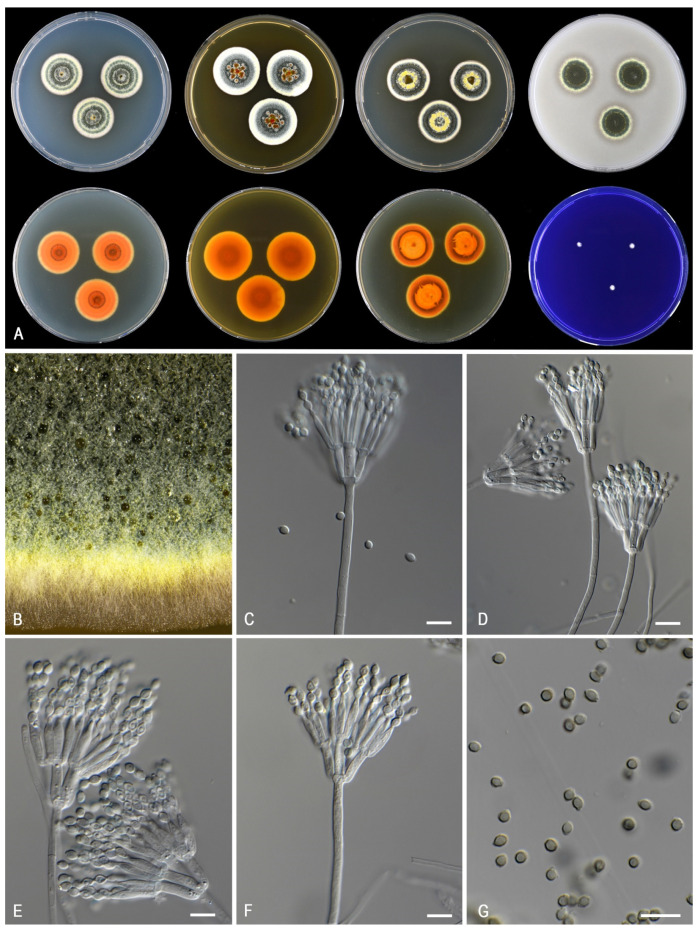
Morphological characters of *Talaromyces calidominioluteus* (DTO 390-E9). (**A**). Colonies, from left to right, after 7 d at 25 °C (top row) CYA, MEA, YES, OA; (bottom row) CYA reverse, MEA reverse, YES reverse, CREA. (**B**). Detail of colony on MEA. (**C**–**F**). Conidiophores and conidia. (**G**). Conidia. Scale bars = 10 µm.

***Talaromyces calidominioluteus*** Houbraken and Pyrri, sp. *nov.* MycoBank MB 841229. Figure 6.

*Etymology*. Referring to the faster growth on CYA incubated at 30 °C than the other species in the *T. minioluteus*-clade.

*Diagnosis:* Colony diameter on CYA incubated at 30 °C (20–)25–29 mm, conidia smooth, broadly ellipsoidal.

*Typus*: the Netherlands (imported from Brazil), melon, isolated by J. Houbraken, Oct. 2007 (holotype CBS H-24875, culture ex-type CBS 147313 = DTO 052-G3).

ITS barcode: OK339612 (alternative markers: *BenA* = OK338786; *CaM* = OK338817; *RPB2* = OK338837).

*Colony diam (7 d, in mm):* CREA 0–6; CYA 20–30; CYA 30 °C (20–)25–29; CYA 37 °C no growth; CYAS 2–10; DG18 15–20; MEA 22–30; OA 17–24; YES 21–29.

*Colony characters: CYA* 25 °C, 7 *d*: Colonies low, slightly sunken in center, plane or with concentric rings, margin entire; sporulation moderate to strong, sometimes poor (DTO 390–E8); conidia *en masse* dark green (26F5–28F3); mycelium pale yellow (2A5–4A5, 1A4–5); colony texture, slightly lanose, sometimes velvety; exudates present as small or large, orange-brown droplets; soluble pigments present, in orange shades (orange, orange-brown), red, or absent; reverse concentric rings of light brown (6D8), then brownish orange (6C6), dark brown (6F8), edge orange (6B6–7). *MEA 25* °C, *7 d:* Colonies low, plane, margin entire; sporulation strong, occasionally moderate; conidia *en masse* grey green (25D5–28E3) to dark green (30F3); mycelium white to pastel yellow (1A4–2A4); colony texture velvety to weakly floccose or cottony; exudates absent or present as orange-brown droplets; soluble pigments absent; reverse orange (6B6–8) to orange brown to brown (6C8–D8). *YES* 25 °C, 7 *d:* Colonies raised in center, concentric and radially sulcate, margin entire; sporulation strong, poor in DTO 390-E8; conidia *en masse* dull green (26E3–4) to dark green (28E–3F3); mycelium pale yellow (1A2–3) to yellow (2A6); exudates absent; soluble pigments absent; reverse orange (6B7) to brownish orange (6C7–7C8). *DG18* 25 °C, 7 *d:* Colonies flat, non-sulcate to radially sulcate, margin entire; sporulation strong (poor in DTO 390-E8); conidia *en masse* dark green (25F8–27F4); mycelium white to yellowish white to pastel yellow (2A2–4); colony texture velvety; exudates absent; soluble pigments absent; reverse yellowish orange (4B8) or greyish orange to orange (6B5–7) or brownish orange (6C8). *OA 25* °C, *7 d*: Colonies plane, low, margins entire; sporulation dense; conidia *en masse* greyish green (27E5–7); mycelium greenish yellow (1A8); colony texture velvety; exudates golden yellow droplets; soluble pigments absent. *CREA* 25 °C, 7 *d*: no growth to poor growth, acid production absent, base formation absent.

*Micromorphology*: Sclerotia and ascomata not observed. Conidiophores biverticillate, occasionally with one subterminal branch or two branches positioned at the same level on the stipe; Stipes smooth-walled, non-vesiculate, 80–200 × 2.5–3.5(–4) µm; Metulae adpressed, 3–10 per stipe, 10–13(–16) × 2.5–3.5 µm; Phialides acerose, 3–8 per metula, 9.5–13.5 × 2–3 µm; Conidia in long, distorted chains, smooth, broadly ellipsoidal, with distinct connectives on both sides, 2.5–4 × 2–2.5 µm.

*Notes*: *Talaromyces calidominioluteus* generally grows faster on CYA incubated at 30 °C (18–29 mm) than the morphologically most similar species *T. minioluteus* (5–11 mm) and *T. gaditanus* (13–20 mm). *Talaromyces africanus* is phylogenetically most closely related and for more details, see description of this species above.

**Figure 7:**
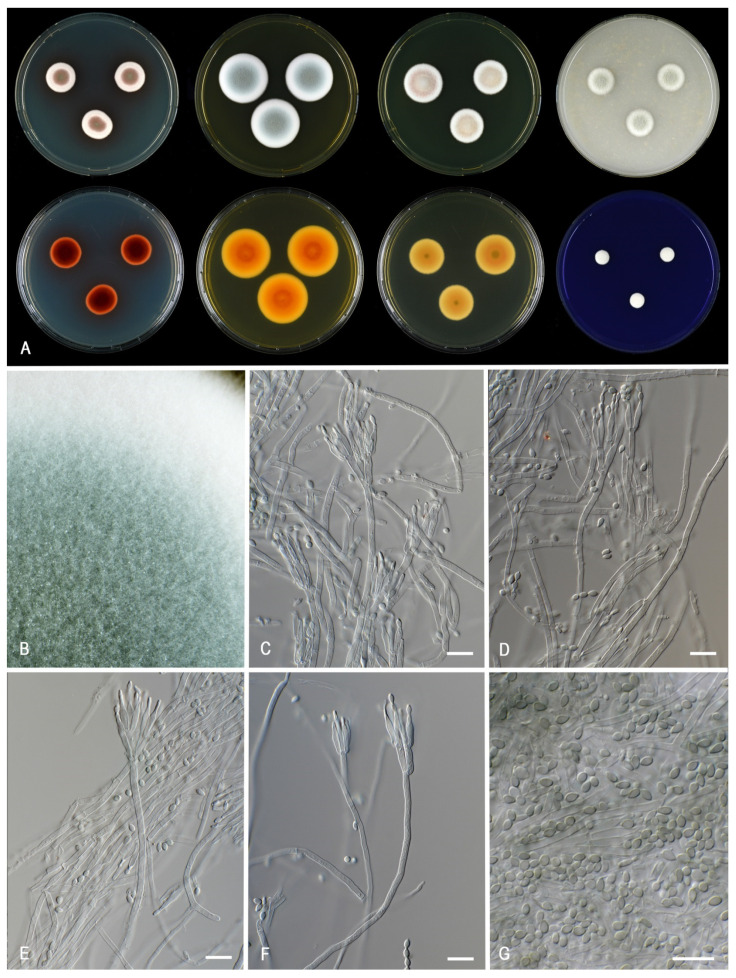
Morphological characters of *Talaromyces chongqingensis* (CBS 147316). (**A**). Colonies, from left to right, after 7 d at 25 °C (top row) CYA, MEA, YES, OA; (bottom row) CYA reverse, MEA reverse, YES reverse, CREA. (**B**). Detail of colony on MEA. (**C**–**F**). Conidiophores and conidia. (**G**). Conidia. Scale bars = 10 µm.

***Talaromyces chongqingensis*** X.C. Wang and W.Y. Zhuang, Biology 10 (8, no. 745): 10. 2021. MycoBank MB 570851. Figure 7.

*Typus*: China, Gaoguan Town, Daba Mountain National Nature Reserve, soil under palm tree, isolated by Xin-Cun Wang, Huan-Di Zheng and Chang Liu, Oct. 2020 (holotype HMAS 247849, culture ex-type CGMCC 3.20482).

ITS barcode: MZ358001 (alternative markers: *BenA* = MZ361343; *CaM* = MZ361350; *RPB2* = MZ361357).

*Colony diam (7 d, in mm):* CREA 7–9; CYA 17–18; CYA 30 °C 6–8; CYA 37 °C no growth; CYAS 3–4; DG18 12–14; MEA 28–29; OA 15–16; YES 20–22.

*Colony characters: CYA* 25 °C, 7 *d*: Colonies plane, moderately deep, margin plane, regular; sporulation poor; conidia *en masse* greenish at the center; mycelium beige; colony texture densely cottony; exudates absent; soluble pigments present, red to dark red (10B–C8); reverse violet brown (10D8), edge red (10B8). *MEA* 25 °C, 7 *d*: Colonies moderately deep, plane, margin entire; sporulation moderate; conidia *en masse* greyish turquoise (24C5); mycelium white; colony texture densely cottony; exudates absent; soluble pigments absent; reverse golden yellow (5B7–8). *YES* 25 °C, 7 *d*: Colonies plane, moderately deep, raised at center, margin plane, high, wide regular; sporulation sparse to absent; conidia *en masse* greyish; reverse orange (5B8); mycelium white; exudates absent; soluble pigments absent; *DG18* 25 °C, 7 *d*: colonies plane, raised at center, plane, margin entire; sporulation poor; conidia *en masse* (5A2); mycelium beige in DTO 060-C9; colony texture cottony to floccose; exudates absent or orange; soluble pigments absent; reverse greyish orange (5B6). *OA* 25 °C, 7 *d*: colonies plane, moderately deep, margin plane, raised, wide, regular; sporulation moderate; conidia *en masse* greenish grey (27C2); mycelium white; colony texture floccose; exudates absent; soluble pigments absent; *CREA* 25 °C, 7 *d*: growth, acid production absent, base formation absent.

*Micromorphology*: Sclerotia and ascomata not observed. Conidiophores biverticillate and several monoverticillate; Stipes smooth walled, non-vesiculate 50–100 × 2.5–3.5 µm; Metulae adpressed, 2–5 per stipe, 11.5–15 × 2.5–3.5 µm; Phialides cylindrical to acerose, 2–5 per metula, 9–12 × 2.5–3.5 µm; Conidia in long, distorted chains, smooth, ovoidal, 2.5–3.5 × 1.5–2.5 µm.

*Notes*: The ex-type strain was not available for examination during our study and the description above is based on CBS 270.35 (=CBS 147316). The phenotype of this old strain, isolated from *Zea mays* (corn) in 1912, differs significantly from the original description of *T. chongqingensis* [17]. We refer to Zhang et al. [17] for the description based on more recently isolated strains. CBS 270.35 produces densely cottony colonies, sporulates poorly and has greyish-blueish colored conidia on MEA. In contrast, the type of *T. chongqingensis* as a floccose texture on MEA, sporulates well and produces greyish green colored conidia [17]. *Talaromyces chongqingensis* and *T. minioluteus* are phylogenetically most closely related (Figure 1, Figure 2 and Figure 3). CBS 270.35 grows slower (6–8 mm) on CYA incubated at 30 °C than *T. minioluteus* (9–14 mm). Furthermore, CBS 270.35 produces mono- and biverticillate conidiophores and ovoidal conidia, in contrast to the biverticillate conidiophores and broadly fusiform or ellipsoidal conidia produced by *T. minioluteus*. Unfortunately, no data is available for ex-type strain (CGMCC 3.20482) on the growth on CYA at 30 °C, a useful character for species identification in the *T. minioluteus*-clade. CBS 270.35 could be degenerated and might not be a good representative of the species. However, differences in the phenotype can also occur when a strain is grown at different labs. In order to make a more reliable comparison of the macromorphology of *T. chongqingensis* with the current data, the freshly isolated ex-type strain (CGMCC 3.20482) should be grown under the same conditions as the other strains in this study.

**Figure 8:**
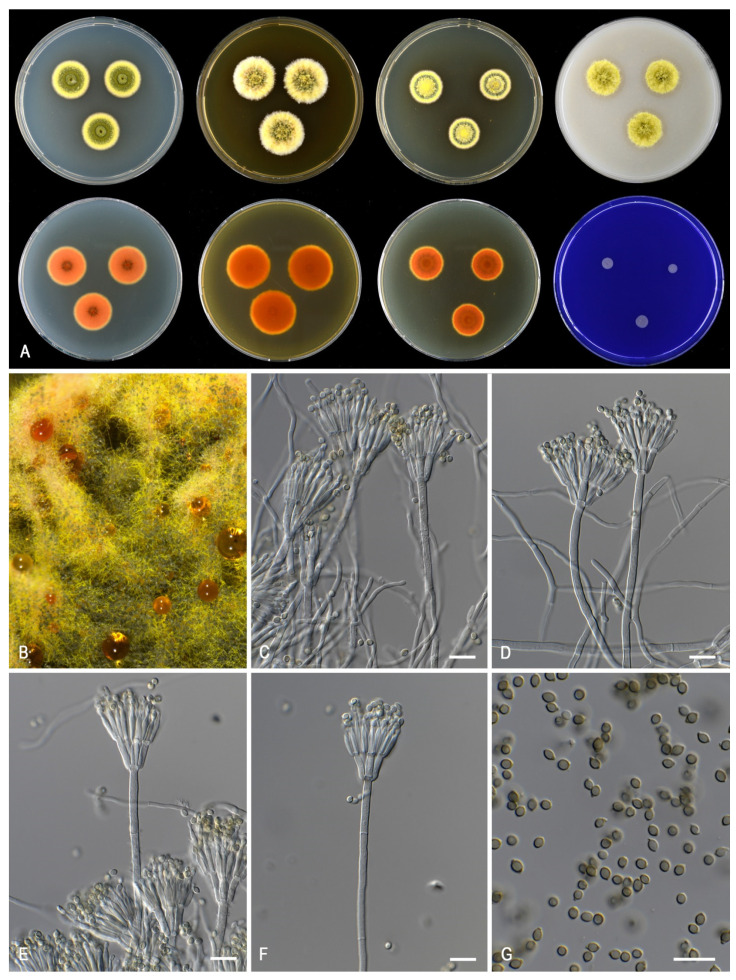
Morphological characters of *Talaromyces gaditanus* (CBS 169.81). (**A**). Colonies, from left to right, after 7 d at 25 °C (top row) CYA, MEA, YES, OA; (bottom row) CYA reverse, MEA reverse, YES reverse, CREA. (**B**). Detail of colony on MEA. (**C**–**F**). Conidiophores and conidia. (**G**). Conidia. Scale bars = 10 µm.

***Talaromyces gaditanus*** (C. Ramírez and A.T. Martínez) Houbraken and Soccio, ***comb. nov.*** MycoBank MB 841226. Figure 8.

*Basionym*: *Penicillium gaditanum* C. Ramírez and A.T. Martínez, Mycopathol. 74: 165. 1981.

*Typus*: Spain, Madrid, air, 1978, isolated by C. Ramírez (holotype IJFM 5146, culture ex-type CBS 169.81 = DTO 228-B8 = ATCC 42230 = IMI 253792 = VKM F-2188 = IJFM 5146).

ITS barcode: MH861318 (alternative markers: *BenA* = OK338775; *CaM* = OK338802; *RPB2* = OK338827).

*Colony diam (7 d, in mm):* CREA 2–6; CYA 19–25; CYA 30 °C 12–16; CYA 37 °C no growth; CYAS 2–6; DG18 10–18; MEA 25–28; OA 15–19; YES 18–25.

*Colony characters: CYA 25* °C, *7 d*: Colonies low, slightly sunken at center, plane, sulcate near center, margin entire; sporulation strong; conidia *en masse* greyish green to dark green (28E7–F7–8); mycelium greenish yellow (1A6–7) to light yellow (2A5–6); colony texture velvety, slightly funiculose; exudates absent; soluble pigments present, orange shade; reverse brown (6D8) at the center, orange (5B8–6B8) elsewhere. *MEA 25* °C, *7 d*: Colonies low, plane, margin entire; sporulation moderate to dense; conidia *en masse* greyish green (28E7), dark green (27F7–9); mycelium pastel yellow (1A4–6, 2A4); colony texture floccose; exudates absent or present as red droplets; soluble pigments absent; reverse brown (6D8), reddish orange (7B8). *YES 25* °C, *7 d*: Colonies raised at center, having a leathery appearance, plane, lightly sulcate to radially sulcate, margin entire; sporulation absent, sparse to moderate; conidia *en masse* light brown (7D8) in center, orange (5B8) in middle, dark green (25E4–5, 26F7–8); mycelium white, pastel yellow (1A4); exudates absent; soluble pigments absent, brownish orange (6C8); reverse yellow (3A6) edge, brownish yellow (5C8) in center, brownish orange to reddish orange (7B8) to brownish red (8C8). *DG18 25* °C, *7 d*: Colonies a little raised at center, plane, margin entire; sporulation poor in DTO 228-B8, dense in other isolates; conidia *en masse* greyish green (28E7, 28C6–7); mycelium pastel yellow (2A4), yellow (2A6) in DTO 228-B8, greenish yellow (1A6) in other isolates; colony texture cottony in DTO 228-B8, velvety with floccose at center; exudates absent, yellow, red in DTO 335-A5; soluble pigments absent; reverse orange (6B7), orange to brownish orange to reddish orange (6B8–7C8). *OA 25* °C, *7 d*: Colonies moderately deep, plane; margin entire; sporulation poor; conidia *en masse* greyish green (27E6); mycelium greenish yellow (1A6); colony texture floccose; exudates absent; soluble pigments reddish. *CREA 25* °C, *7 d*: poor growth, acid production absent, base formation absent.

*Micromorphology*: Sclerotia and ascomata not observed. Conidiophores biverticillate; Stipes smooth-walled, non-vesiculate, 80–200 × 2.5–3.5 µm; Metulae adpressed, 3–8 per stipe, 10–13.5 × 2.5–3.5 µm; Phialides acerose, 3–8 per metula, 8.5–12.5 × 1.5–3 µm; Conidia in long, distorted chains, smooth, fusiform, with distinct connectives on both sides, 2.5–4 × 2–2.5(–3) µm.

*Notes*: This species was originally described as *Penicillium gaditanum*. Based on morphological and chemical data, *Penicillium gaditanum* was treated by van Reenen-Hoekstra et al. [3] and Yilmaz et al. [4] as a synonym of *T. minioluteus*. A molecular treatment of this species was lacking and sequence analyses presented here shows that this species is related to *T. minioluteus*, but distinct based on the GCPSR concept. This species produces strongly floccose colonies on MEA, with bright yellow (pastel yellow) mycelium and fusiform, smooth-walled conidia. Phenotypically, this species resembles *T. samsonii*. *Talaromyces samsonii* has a violet brown to deep red reverse on CYA and (vivid) red soluble pigments, in contrast to the predominantly orange reverse and orange soluble pigments (if present) in *T. gaditanus*.

**Figure 9:**
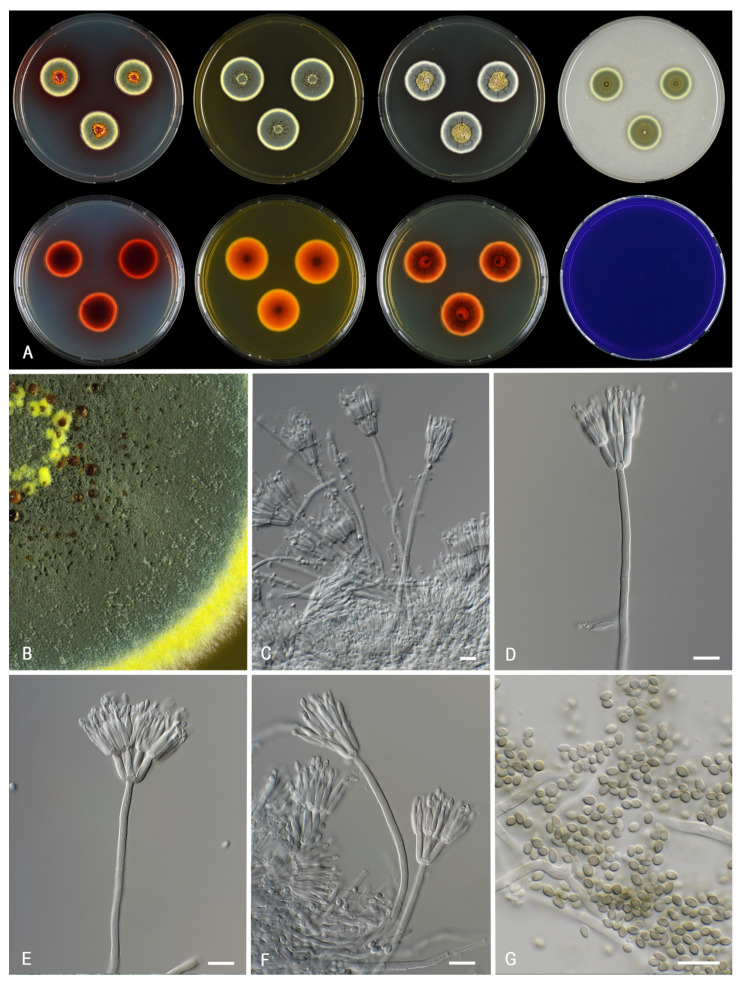
Morphological characters of *Talaromyces germanicus* (CBS 147314). (**A**). Colonies, from left to right, after 7 d at 25 °C (top row) CYA, MEA, YES, OA; (bottom row) CYA reverse, MEA reverse, YES reverse, CREA. (**B**). Detail of colony on MEA. (**C**–**F**). Conidiophores and conidia. (**G**). Conidia. Scale bars = 10 µm.

***Talaromyces germanicus*** Houbraken and Pyrri, sp. *nov*. MycoBank MB 841227. Figure 9.

*Etymology*. Referring to Germany, the country where the type was isolated.

*Diagnosis:* Colony diameter on CYA incubated at 30 °C 6–7 mm, colony texture on MEA velvety, conidial color *en masse* on MEA dull green to greyish green.

*Typus*: Germany, drywall (wallboard), July 2009 (holotype CBS H-24876, culture ex-type CBS 147314 = DTO 055-D1).

ITS barcode: OK339619 (alternative markers: *BenA* = OK338799; *CaM* = OK338812; *RPB2* = OK338845).

*Colony diam (7 d, in mm):* CREA no growth; CYA 20–22; CYA 30 °C 6–7; CYA 37 °C no growth; CYAS no growth; DG18 16–18; MEA 23–25; OA 18–19; YES 22–24.

*Colony characters: CYA 25* °C, *7 d:* Colonies radially sulcate with a concentric ring, margin plane, low, moderately wide, regular; sporulation dense; conidia *en masse* dull green to greyish green (26D4–5); mycelium pastel yellow (1A4); colony texture velvety; exudates cherry red (10B8) only small drops at the center; soluble pigments present, cherry red (10B8); reverse violet brown (10F7) centrally, brownish red (10D7) elsewhere. *MEA 25* °C, *7 d*: Colonies low, with a few sulcations near the center, margin plane, low, wide, regular; sporulation dense; conidia *en masse* dull green to greyish green (25E4–5); mycelium light yellow (1A6); colony texture velvety; exudates red; soluble pigments absent; reverse reddish orange (7B8). *YES 25* °C, *7 d:* Colonies radially sulcate, raised and densely sulcate at the center, margin plane, low, moderate, regular; sporulation dense; conidia *en masse* dull green to greyish green (25D4–5); mycelium pastel yellow (1A4); exudates golden; soluble pigments tomato red (8C8); reverse brownish red (9C8). *DG18 25* °C, *7 d:* colonies plane, low, margin plane, low, moderate, regular; sporulation dense; conidia *en masse* dark green (25F7–8); mycelium white; colony texture velvety; exudates absent; soluble pigments absent; reverse copper red (7C8) at the center, orange (5B8) elsewhere. *OA 25* °C, *7 d:* colonies plane, low, margin plane, low, moderate, regular; sporulation dense; conidia *en masse* greyish green (27E5–28E5); mycelium light yellow (1A6); colony texture velvety; exudates absent; soluble pigments absent. *CREA 25* °C, *7 d*: no growth.

*Micromorphology*: Sclerotia and ascomata not observed. Conidiophores biverticillate; Stipes smooth-walled, non-vesiculate, 90–150 × 2.5–3 µm; Metulae adpressed, 4–6 per stipe, 11.5–15 × 2.5–3.5 µm; Phialides acerose, 4–6 per metula, 11–15.5 × 2–3 µm; Conidia in long, distorted chains, smooth, narrow ellipsoidal to slightly fusiform, 2.5–3.5(–4) × 1.5–2.5 µm.

*Notes*: *Talaromyces germanicus* grows slower on CYA, MEA and YES than the phylogenetically related *T. minnesotensis*. *Talaromyces germanicus*, *T. chongqingensis* and *T. udagawae* have similar growth rates on CYA incubated at 30 °C. The first two species can be differentiated by their colony texture and conidial color on MEA: *T. germanicus* produces velvety colonies with dull green to greyish green conidia, in contrast to the floccose colonies with greyish turquoise conidia of *T. chongqingensis*. *Talaromyces udagawae* produces a sexual state, a feature not observed in the other *T. minioluteus*-clade species.

**Figure 10:**
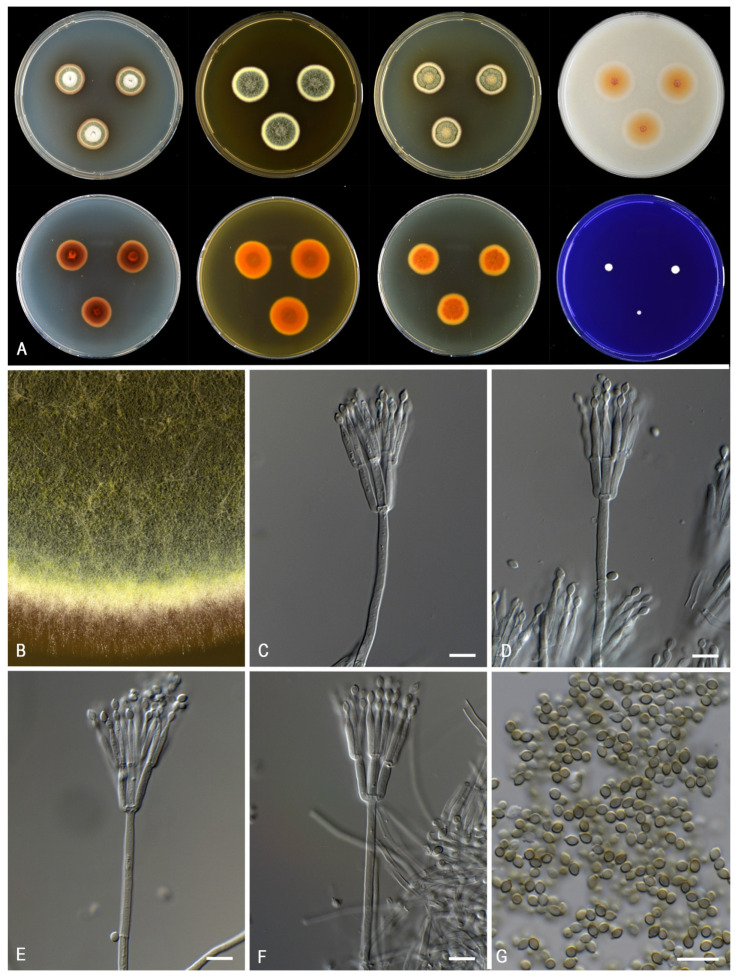
Morphological characters of *Talaromyces minioluteus* (CBS 642.68). (**A**). Colonies, from left to right, after 7 d at 25 °C (top row) CYA, MEA, YES, OA; (bottom row) CYA reverse, MEA reverse, YES reverse, CREA. (**B**). Detail of colony on MEA. (**C**–**F**). Conidiophores and conidia. (**G**). Conidia. Scale bars = 10 µm.

***Talaromyces minioluteus*** (Dierckx) Samson et al., Stud. Mycol. 70: 176. 2011. MycoBank MB 560657. Figure 10.

*Basionym*: *Penicillium minioluteum* Dierckx, Ann. Soc. Sci. Bruxelles 25: 87. 1901. MycoBank MB 157378.

*Neotype*: Unknown location and substrate (neotype IMI 89377ii [Pitt et al., 1980], culture ex-neotype CBS 642.68 = DTO 304-C4 = DTO 037-B6 = CCRC 31698 = IMI 089377 = LSHB P44 = MUCL 28666 = NRRL 1714).

ITS barcode: JN899346 (alternative markers: *BenA* = MN969409; *CaM* = KJ885273; *RPB2* = JF417443).

*Colony diam (7 d, in mm):* CREA 4–8; CYA 17–19; CYA 30 °C 9–14; CYA 37 °C no growth; CYAS no growth or 3–4; DG18 11–16; MEA 21–26; OA 9–16; YES 16–22.

*Colony characters: CYA 25* °C, *7 d*: Colonies low, raised at center, radially sulcate towards margins, margin entire; sporulation moderate to dense in a ring towards the margins; conidia *en masse* greyish green (27C4–5); mycelium yellowish; colony texture velvety to weakly floccose; exudates absent; soluble pigments present, light brow (6D8), brownish orange (7C6–8); reverse light brown (6D8) in center, dark brown (6F8) in a ring under dense sporulation, edge sometimes orange (6B8). *MEA 25* °C, *7 d*: Colonies moderately deep, plane, margin entire; sporulation strong; conidia *en masse* dark green (27F7–8); mycelium pastel yellow (1A4); colony texture loosely funiculose; exudates absent; soluble pigments absent; reverse brownish orange (6C8), at margin orange (6B8). *YES 25* °C, *7 d*: Colonies slightly radially sulcate, umbonate, moderately deep, raised in center, margin entire; sporulation strong; conidia *en masse* dull green (27D4–E4); mycelium beige (4A3); exudates absent; soluble pigments orange to brownish orange (6B8–C8); reverse orange to brownish orange (6B8–C8). *DG18 25* °C, *7 d*: colonies plane, raised in center, plane, margin entire; sporulation moderate to strong; conidia *en masse* deep green (27D8–E8) or dull green (27D3); mycelium yellowish (2A2); colony texture cottony to floccose; exudates absent or orange; soluble pigments absent; reverse brownish orange to light brown in center (7C8–D8), margins reddish to brownish orange (7B8–C8). *OA 25* °C, *7 d*: colonies plane, low, margin entire; sporulation strong; conidia *en masse* dull green to greyish green (27D3–E3–5); mycelium beige; colony texture cottony; exudates absent; soluble pigments absent. *CREA 25* °C, *7 d*: no growth.

*Micromorphology*: Sclerotia and ascomata not observed. Conidiophores biverticillate, occasionally with an additional branch; Stipes smooth-walled, non-vesiculate, 100–250 × 2.5–3.5 µm; Metulae adpressed, 2–8 per stipe, 11–15.5 × 2.5–3.5 µm; Phialides acerose, 3–8 per metula, 10.5–15.5 × 2–2.5 µm; Conidia in long, distorted chains, smooth, broadly ellipsoidal or ellipsoidal, sometimes with a distinct connective, 2.5–3.5 × 1.5–2.5 µm.

*Notes*: *Talaromyces minioluteus* is phylogenetically related to *T. gaditanus*, *T. chongqingensis* and *T. samsonii*. These species have, with the exception of *T. chongqingensis*, a similar growth rate on CYA 30 °C, CYA and MEA. Colonies of *T. samsonii* are more densely sporulating and have a floccose texture on MEA while *T. minioluteus* sporulates moderately dense and has a strongly funiculose texture. The colony reverse and soluble pigments produced by *T. minioluteus* are in shades of brown (light brown, dark brown, orange-brown), while the reverse of *T. samsonii* is violet-brown to deep red and the soluble pigments vivid red to red.

**Figure 11:**
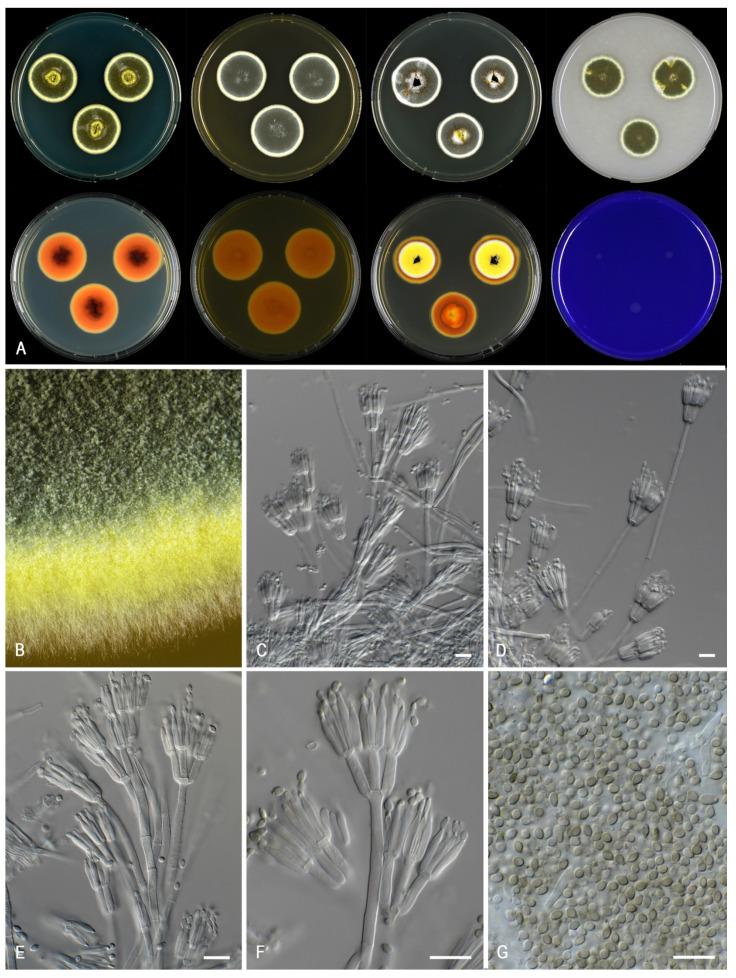
Morphological characters of *Talaromyces minnesotensis* (CBS 141838). (**A**). Colonies, from left to right, after 7 d at 25 °C (top row) CYA, MEA, YES, OA; (bottom row) CYA reverse, MEA reverse, YES reverse, CREA. (**B**). Detail of colony on MEA. (**C**–**F**). Conidiophores and conidia. (**G**). Conidia. Scale bars = 10 µm.

***Talaromyces minnesotensis*** Guevara-Suarez et al., Mycoses 60: 657. 2017. MycoBank MB 820463. Figure 11.

*Typus*: USA, Minnesota, from human ear, 2010, isolated by D.A. Sutton (holotype CBS H-23001, culture ex-type CBS 142381 = DTO 423-A7 = UTHSC DI16-144 = FMR 14265).

ITS barcode: LT558966 (alternative markers: *BenA* = LT559083; *CaM* = LT795604; *RPB2* = LT795605).

*Colony diam (7 d, in mm):* CREA 0–5; CYA 20–24; CYA 30 °C 20–24; CYA 37 °C no growth; CYAS 0–9; DG18 14–19; MEA 28–30; OA 18–22; YES 19–28.

*Colony characters: CYA 25* °C, *7 d*: Colonies low, radially sulcate with concentric rings, crateriform, margin entire, plane low, moderate, regular; sporulation dense; conidia *en masse* dull green to dark green (27E6–F6); mycelium pastel yellow (1A4); colony texture velvety; exudates absent; soluble pigments absent; reverse dark brown (8F7) at the center, reddish orange (7A7) elsewhere. *MEA 25* °C, *7 d*: Colonies plane or lightly sulcate near the center, low to moderately deep, margin entire, plane, low, moderate to wide; sporulation strong;); conidia *en masse* dull green to greyish green (25D4–E4–5); mycelium white to pastel yellow (1A4); colony texture velvety; exudates absent; soluble pigments absent or (10B8−C8reverse orange (5B8) or violet brown (10F8). *YES 25* °C, *7 d*: Colonies raised at center, lightly sulcate at the center to radially sulcate and sulcate at the center, margin entire, plane, low, moderate, regular; sporulation dense; conidia *en masse* greyish green (25D4–5) to greenish grey (27E2–3); mycelium white or pale yellow (1A3); exudates absent to golden red; soluble pigments absent or (9B6); reverse dark red (11C8) at the center with a greyish red (11D5) ring near the margins or copper red (7C8) with a brown (7F4) ring near center. *DG18 25* °C, *7 d*: Colonies plane, low, margin entire, plane, low, moderate, regular; sporulation dense; conidia *en masse* greyish green to dark green (27E5–F7); mycelium white (2A2–4); colony texture velvety; exudates absent; soluble pigments clear or absent; reverse orange (5B8−6B8) or corn to amber yellow (4B5–6) with a golden yellow (5B7) ring near center. *OA 25* °C, *7 d*: Colonies plane, low; margin entire, plane, low, moderate to wide, regular; sporulation moderate to dense; conidia *en masse* dull green (27D3–E3) or greyish green (27E5–7); mycelium white to greenish yellow (1A7); colony texture velutinous to granulose; exudates absent to dark red (11C8); soluble pigments absent. *CREA 25* °C, *7 d*: no growth to poor growth, acid production absent, base formation absent.

*Micromorphology*: Sclerotia and ascomata not observed. Conidiophores biverticillate; Stipes smooth-walled, non-vesiculate, 100–250 × 2–3 µm; Metulae adpressed, 3–5 per stipe, 9.5–15.5 × 2.5–3.5 µm; Phialides acerose, 3–5 per metula, 11–15.5 × 2–3 µm; Conidia in long, distorted chains, smooth, ellipsoidal, sometimes with distinct connectives, 2.5–3.5(–4.5) × 1.5–2.5 µm.

*Notes*: *Talaromyces minnesotensis* is phylogenetically related to *T. germanicus*. The former species grows faster on CYA incubated at 30 °C.

**Figure 12:**
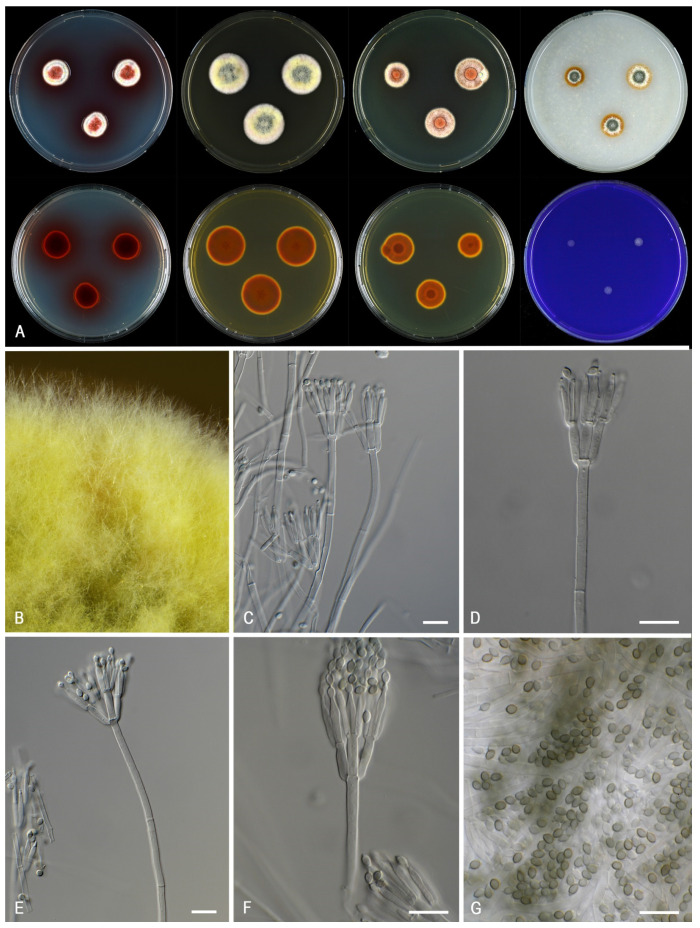
Morphological characters of *Talaromyces samsonii* (CBS 137.84). (**A**). Colonies, from left to right, after 7 d at 25 °C (top row) CYA, MEA, YES, OA; (bottom row) CYA reverse, MEA reverse, YES reverse, CREA. (**B**). Detail of colony on MEA. (**C**–**F**). Conidiophores and conidia. (**G**). Conidia. Scale bars = 10 µm.

***Talaromyces samsonii*** (Quintan.) Houbraken and Pyrri, *comb. nov.* MycoBank MB 841230. Figure 12.

*Basionym*: *Penicillium samsonii* Quintan. Mycopathol. 91: 69. 1985.

*Typus*: Lectotype designated here: CBS H-24877, MBT 10002955. Spain, Valladolid; apple (*Malus sylvestris*) damaged by insect, isolated by J.A. Quintanilla (culture ex-type CBS 137.84 = DTO 304-C3 = DTO 169-G6 = CECT 2772 = IMI 282404 = IMI 327872).

ITS barcode: MH861709 (alternative markers: *BenA* = OK338798; *CaM* = OK338824; *RPB2* = OK338844).

*Colony diam (7 d, in mm):* CREA 3–4; CYA 15–21; CYA 30 °C 11–16; CYA 37 °C no growth; CYAS 4–5; DG18 14–17; MEA 22–26; OA 12–15; YES 15–25.

*Colony characters: CYA 25* °C, *7 d*: Colonies slightly sulcate, slightly raised at center, margin plane, low, moderate, regular; sporulation moderate to dense; conidia *en masse* greyish green to dark green (27B3–5, 27F6–8); mycelium sulfur yellow (1A5); colony texture velvety to velvety to weakly floccose; exudates absent; soluble pigments present, vivid red to red (10A8−B8); reverse violet brown to deep red (10E8−F8, 7B8−C8). *MEA 25* °C, *7 d*: Colonies moderately deep, slightly radially sulcate, margin entire; sporulation strong; conidia *en masse* dark green (27F7–8, 28E4–5); mycelium light yellow to sulfur yellow (1A4–5); colony texture floccose; exudates absent; soluble pigments absent; reverse copper red to red (7B8−C8−D8). *YES 25* °C, *7 d*: Colonies slightly radially sulcate, umbonate, raised in center, margin entire; sporulation strong; conidia *en masse* dull green to greyish green (26D4–5); mycelium pastel yellow (2A4); exudates absent; soluble pigments orange to brownish orange (6B8−C8); reverse reddish brown to cherry red (8D8, 10B8) at the center, blood red (10C8−D8) elsewhere. *DG18 25* °C, *7 d*: colonies plane, raised in center, margin plane, flat, wide, regular; sporulation moderate to strong; conidia *en masse* greyish green to deep green (27E6–8); mycelium yellowish (2A2); colony texture cottony to floccose; exudates absent; soluble pigments absent; reverse brownish orange to light brown in center (7C8–D8–9), margins reddish to brownish orange (7B8–C8). *OA 25* °C, *7 d*: colonies plane, low, margin entire; sporulation strong; conidia *en masse* dull green to greyish green (27E3–5) to dark green (27F6–8) at the center, greyish green (27C5) elsewhere; mycelium light yellow (1A4); colony texture velvety to cottony; exudates absent; soluble pigments absent. *CREA 25* °C, *7 d*: no growth.

*Micromorphology*: Sclerotia and ascomata not observed. Conidiophores biverticillate, occasionally with an additional branch stipes smooth-walled, non-vesiculate, 90–200 × 2.5–3.5 µm wide. Metulae adpressed, 2–8 per stipe, 10.5–20.5(–23) × 2.0–4.5 µm. Phialides acerose, 3–8 per metula, 9.5–17 × 2–3.5 µm. Conidia in long, distorted chains, smooth, ellipsoidal to fusiform, sometimes with a distinct connective, 2.5–4(–5) × 1.5–3 µm.

*Notes*: See also *T. minioluteus*. No holotype material of *P. samsonii* was deposited in a herbarium. A strain with a personal code (1032) is mentioned in the protologue and subcultures of this strain were deposited in CBS, IMI and CECT [15]. A subculture present in the CBS collection (CBS 137.84) is deposited in the CBS herbarium as CBS H-24877 and used as lectotype.

**Figure 13:**
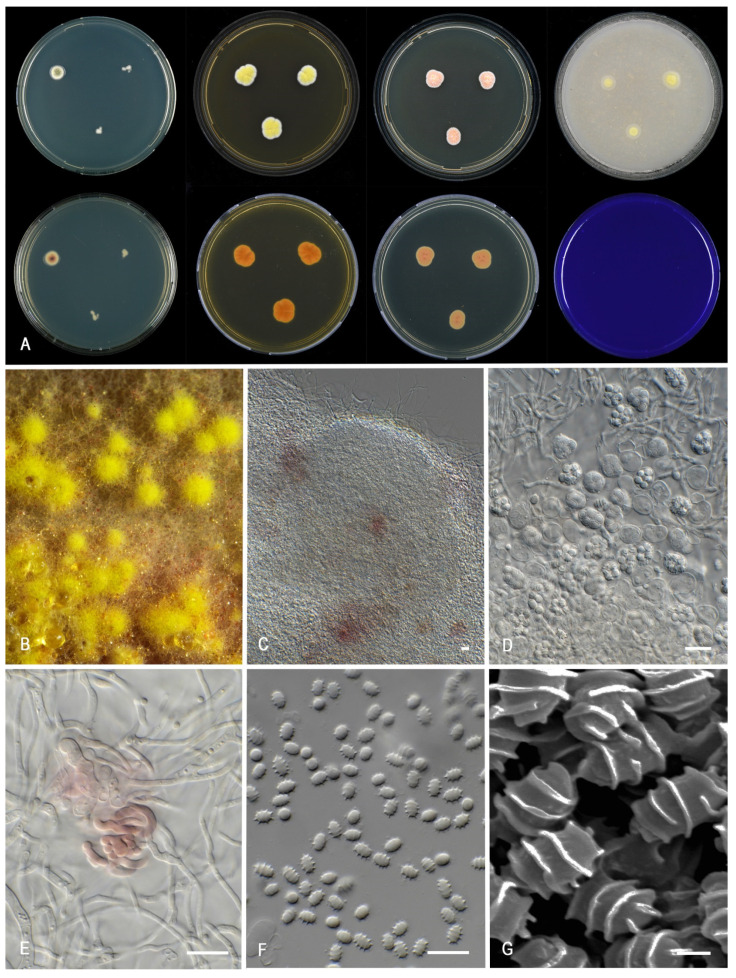
Morphological characters of *Talaromyces udagawae* (CBS 579.72). (**A**). Colonies, from left to right, after 7 d at 25 °C (top row) CYA, MEA, YES, OA; (bottom row) CYA reverse, MEA reverse, YES reverse, CREA. (**B**). Detail of colony on MEA. (**C**). Ascoma. (**D**). Asci and ascospores. (**E**). Initial. (**F**,**G**). Ascospores. Scale bars = 10 µm; except (**G**), 1 µm.

***Talaromyces udagawae*** Stolk and Samson, Stud. Mycol. 2: 36. 1972. MycoBank MB 324424. Figure 13.

≡*Penicillium udagawae* Stolk and Samson, Stud. Mycol. 2: 36. 1972.

*Typus*: Japan, Misugimura, soil, isolated by S. Udagawa, 1964 (holotype CBS H-7841, culture ex-type CBS 579.72 = DTO 302-A8 = FRR 1727 = IFO 8808 = IMI 197482 = NHL 6089).

ITS barcode: JN899350 (alternative markers: *BenA* = OK338783; *CaM* = MH861709; *RPB2* = MN969148).

*Colony diam (7 d, in mm):* CREA no growth; CYA 5–9; CYA 30 °C 5–8; CYA 37 °C no growth; CYAS no growth; DG18 3–4; MEA 15–19; OA 13–17; YES 11–14.

*Colony characters*: *CYA 25* °C, *7 d*: Colonies plane, raised at the center, margin plane, low, regular; sporulation absent; conidia *en masse* green dull green (28D4); mycelium white; colony texture floccose; exudates absent; soluble pigments absent, vivid red to cherry red (10A8−B8); reverse brownish orange (5C5). *MEA 25* °C, *7 d*: Colonies plane, low, margin plane, low, entire; sporulation absent; conidia *en masse* greyish green (25D5); mycelium pastel yellow (1A4); colony texture floccose; exudates absent; soluble pigments absent; reverse mandarin orange (6B8). *YES 25* °C, *7 d*: Colonies plane with a concentric ring, low, margin plane, low, entire; sporulation absent; conidia *en masse* dull green (25E4); mycelium white; exudates absent; soluble pigments absent; reverse greyish yellow (2C4). *DG18 25* °C, *7 d*: Colonies plane, low, margin plane, low, regular; sporulation absent; conidia *en masse* white; mycelium white; colony texture floccose; exudates absent; soluble pigments absent; reverse yellowish. *OA 25* °C, *7 d*: colonies plane, low, margin entire; sporulation strong; conidia *en masse* greyish green to jade green (27E5–7); mycelium yellow (1A6); colony texture velvety; exudates absent; soluble pigments absent. *CREA 25* °C, *7 d*: no growth.

*Microscopy*: Asexual state *fide* Stolk and Samson [31]: Sclerotia not observed. Conidiophores biverticillate; Stipes smooth-walled, non-vesiculate, 50–200 × 2.5–3.5 µm; Metulae divergent, 2–5 per stipe, 7.5–10 × 2.0–2.5 µm; Phialides acerose, 3–6 per metula, 12–15 × 2–2.5 µm; Conidia smooth, subglobose to ellipsoidal, 3–4 × 2–3 µm; Ascomata after 2–3 wk, globose to subglobose, 200–400 μm; Asci 8–13.5 × 6–9.5 μm; Ascospores ellipsoidal, ornamented with three to five regular, nearly parallel, transverse ridges, often spirally arranged, 3.5–6 × 2.5–3.5 μm.

*Notes*: *Talaromyces udagawae* can be distinguished from other *T. minioluteus*-clade species by the production of ascomata and its distinctive ascospores, which are ornamented with three to five regularly transverse, nearly parallel ridges. It has restrictive growth on media examined and does not grow on CREA, DG18 or CYA incubated at 37 °C. The asexual morph was not observed during this study, but was described in Stolk and Samson [31].

## 4. Discussion

Visagie et al. [18] suggested that *T. minioluteus* could represent a species complex, and more recently, two species were described in this complex, *T. chongqingensis* and *T. minnesotensis* [16,17]. In addition to the ex-neotype strain of *T. minioluteus* (CBS 642.68), also the ex-type strains of *P. samsonii* (CBS 137.84) and *P. purpurogenum* var. *rubisclerotium* (CBS 270.35) belong to this clade [4]. Based on morphological similarities and sequence data (ITS and *BenA*), Yilmaz et al. [4] considered the latter two species synonyms of *T. minioluteus*. *Penicillium gaditanum* (CBS 169.81) was not included in their phylogenetic analyses and this species was treated as synonym of *T. minioluteus* based on morphological and chemical data [3]. In our study, we show that both *P. gaditanum* and *P. samsonii* are distinct species and we provide new *Talaromyces* combinations for these.

The taxonomic position of *P. purpurogenum* var. *rubrisclerotium* was uncertain. Yilmaz et al. [13] noted that many strains previously identified as *P. purpurogenum* var. *rubrisclerotium* resolved in a clade with *T. amestolkiae*, but that its presumed ex-type strain CBS 270.35 clustered with *T. minioluteus* [4]. Our results indicate that CBS 270.35 is in fact *T. chongqingensis*. Thom [32] reported strain Thom 2670 as representative of *P. purpurogenum* var. *rubrisclerotium*, producing sclerotia and able to grow at 37 °C. Both CBS 270.35 and CGMCC 3.20482 (ex-type of *T. chongqingensis*) deviate from the original description of *P. purpurogenum* var. *rubrisclerotium* [32] by lacking sclerotia production and their inability to grow at 37 °C [17]. Raper and Thom [12], Pitt [5] and Peterson and Jurjević [33] considered NRRL 1064 as the ex-type of *P. purpurogenum* var. *rubrisclerotium*. Raper and Thom [12] mentioned that there was little to suggest that NRRL 1064 was representative of the original concept of *P. purpurogenum* var. *rubrisclerotium*. This strain morphological deviates from the original description in being floccose, poorly sporulating and lacking production of dark-red sclerotia, which is a hallmark character of *P. purpurogenum* var. *rubrisclerotium* [5]. Based on morphology, Pitt [5] classified NRRL 1064 under *Penicillium pinophilum*. Using sequence data, Peterson and Jurjević [33] showed that NRRL 1064 phylogenetically represents *T. sayulitensis*, a close relative of *T. pinophilus*. Both NRRL 1064 and CBS 138204 (ex-type of *T. sayulitensis*) grow well on CYA at 37 °C [5,18], while CBS 270.35 and CGMCC 3.20482 don’t ([4], this study). There is a chance that both NRRL 1064 and CBS 270.35 were contaminated in the past. Nevertheless, based on the ability to grow at 37 °C, we consider NRRL 1064 as the ex-type of *P. purpurogenum* var. *rubrisclerotium* and treat this variety as a synonym of *T. sayulitensis* (classified in sect *Talaromyces*), a species phylogenetically unrelated to *T. minioluteus*. Because a name has no priority outside the rank in which it is published, the name *P. purpurogenum* var. *rubrisclerotium* does not compete with *T. sayulitensis* (Figure 1).

The *T. minioluteus*-clade strains studied here share macro- and micromorphological characters. None of the species can grow on CYA incubated at 37 °C and all are able grow at 30 °C, though with different growth rates. They grow more restricted on CYA incubated at 25 °C than on MEA and generally form colonies having greyish green conidia with a velvety, floccose or funiculose texture. Most of the strains produce reddish pigments on CYA and/or MEA. The strains investigated in our study did not or poorly grew on CREA and they did not produce acid compounds, although Guevara-Suarez et al. [16] reported acid production in *T. minnesotensis*. The *T. minioluteus*-clade species can be differentiated using phenotypic characters and an overview of the most important characters is given in Table 2. The colony diameter on CYA incubated at 30 °C, colony texture on MEA, and conidial shape and ornamentation proved to be the most useful characters for identification. No sexual morph was observed in any of the *T. minioluteus*-clade strains. The closely related species *T. udagawae* forms ascomata that contain ascospores with almost parallel ridges [4,31,34]. The asexual morph was not observed in our study, but Stolk and Samson [31] observed this morph on hay-infusion agar. The conidiophores of this species are symmetrically biverticillate with long metulae.

In total, eight species are resolved in the *T. minioluteus*-clade based on this polyphasic approach combining morphology and physiology with sequence data of four loci (*BenA*, *CaM*, ITS and *RPB2*). The topologies of the single gene phylogenies are similar and largely adhere to the genealogical concordance phylogenetic species recognition (GCPSR) concept [35]. The exception is the phylogenetic relationship of CBS 147336 (=DTO 162-E5). This strain clusters with *T. minnesotensis* in the *BenA*, *CaM* and ITS phylogenies, but has a different position in the *RPB2* phylogram, where it is a sister of *T. calidominioluteus* and *T. africanus* (80% BS, 0.98 pp). The morphology of CBS 147336 differs from the examined *T. minnesotensis* strains and may represent a new taxon (Figure 4). Macromorphologically, CBS 147336 grows slower on all culture media and unlike *T. minnesotensis*, it does grow on CREA. It produces greenish grey colored conidia, white mycelium and red pigments whereas *T. minnesotensis* has deep green conidia, yellow mycelium and orange pigments. *Talaromyces minnesotensis* produces more compact conidiophores with more metulae per stipe and the conidia are slightly smaller in size. To date, there are no new *Talaromyces* species described that share *BenA* and *CaM* sequences with known species, and only differ in their *RPB2* sequence [9]. The remarkable position of this strain could also be due to incomplete lineage sorting or occasional hybridization and introgression, which is also observed in the related genus *Aspergillus* (section *Fumigati*) [36]. Delimitation techniques based on coalescent theory and a multispecies coalescent analysis might be better to deal with this phenomenon than the relatively subjective evaluation and comparison of single-gene trees [36]. We defer with the description of this strain as a new species until more insight is obtained (e.g., by comparative genomics) and preferably, until more strains with this unique *BenA*/*CaM*/ITS/*RPB2* sequence signature are obtained.

All *T. minioluteus* and related species can be identified using *BenA*, *CaM*, *RPB2* or ITS sequences. In order to get insight in the distribution of the investigated species, re-identification of isolates/strains based on *BenA* and ITS sequences present in GenBank showed that 22 of 26 *T. minioluteus* strains/isolates actually represent other taxa in the clade (Supplementary Figure S1, Supplementary Table S1). The majority of sequences clustered with *T. gaditanus* (n = 7) followed by *T. calidominioluteus* (n = 6). In our study, we also found that these two species most common in the *T. minioluteus*-clade. *Talaromyces minioluteus* and related species have a worldwide distribution, but seem to be more frequently encountered in warmer climates [1,37,38]. Furthermore, these species occur on various substrates (e.g., indoor environment, wall paintings and fruits such as citrus, pear and quince). Based on our data and literature review, *T. calidominioluteus* is a postharvest pathogen of quince, tomato and orange fruits [1] and/or is associated with cacao, air/dust and grapes.

Re-evaluation of strains previously identified as *T. minioluteus* or *T. minnesotensis* showed that this clade consists of eight phenotypically similar species. Based on ITS sequences deposited in GenBank, it can be concluded that there is likely more diversity in this clade (Supplementary Figure S1) and a more robust sampling is needed. The present study provides a starting point for future studies on the ability of these species to produce (species-specific) extrolites (incl. mycotoxins). The results of such studies will also give insight in the possible mycotoxin production in foods, as these species are also known as post-harvest pathogens of fruits. Most importantly, this paper provides a more stable taxonomy for a complex that for a long time was problematic.

## Figures and Tables

**Table 1:** Overview of strains used in study.

Species Name	Section	Strain Numbers	Location, Substrate	GenBank Accession Number ^1^			
				BenA	CaM	RPB2	ITS
*Talaromyces calidominioluteus*	*Trachyspermi*	DTO 039-I2 = CBS 113167 = IBT 18572	Unknown location; air in cake factory	**OK338785**	**OK338816**	**OK338836**	**OK339611**
*T. calidominioluteus*	*Trachyspermi*	DTO 052-G3 = CBS 147313	Imported from Brazil to the Netherlands; melon; type of *T. calidominioluteus*	**OK338786**	**OK338817**	**OK338837**	**OK339612**
*T. calidominioluteus*	*Trachyspermi*	DTO 265-H8	Iran, Ajabshir; grapevine	**OK338787**	KU711894	**OK338838**	**OK339601**
*T. calidominioluteus*	*Trachyspermi*	DTO 265-I2 = CBS 147341	Iran, Bonab; grapevine	**OK338788**	KU711896	**OK338839**	**OK339602**
*T. calidominioluteus*	*Trachyspermi*	DTO 266-A5	Iran, Malekan; grapevine	**OK338789**	KU711900	**OK338840**	**OK339603**
*T. calidominioluteus*	*Trachyspermi*	DTO 269-H1	Thailand; house dust	**OK338790**	**OK338818**	**OK338841**	**OK339613**
*T. calidominioluteus*	*Trachyspermi*	DTO 270-A5 = CBS 147342	Thailand; house dust	KP330045	**OK338815**	**OK338835**	**OK339600**
*T. calidominioluteus*	*Trachyspermi*	DTO 390-E9	Nigeria, Ibadan; cocoa beans	MN787900	MN787896	**OK338847**	MN788104
*T. calidominioluteus*	*Trachyspermi*	DTO 390-F1	Nigeria, Ibadan; cocoa beans	MN787901	MN787895	**OK338848**	MN788103
*T. calidominioluteus*	*Trachyspermi*	DTO 390-I9	Nigeria, Ibadan; cocoa beans	MN787911	MN787885	**OK338849**	MN788115
*T. calidominioluteus*	*Trachyspermi*	DTO 391-A5	Nigeria, Ibadan; cocoa beans	MN787914	MN787883	**OK338850**	MN788111
*T. gaditanus*	*Trachyspermi*	CBS 138.84 = CECT 2773 = IMI 282405	Spain, Valladolid; apple (*Malus sylvestris*) damaged by insect	**OK338791**	**OK338819**	**OK338851**	**OK339604**
*T. gaditanus*	*Trachyspermi*	DTO 226-A9 = CBS 104.71	The Netherlands, Lisse; tulip	**OK338792**	**OK338820**	**OK338852**	**OK339614**
*T. gaditanus*	*Trachyspermi*	DTO 226-B1 = CBS 996.72	The Netherlands; jute sugar bag	**OK338774**	**OK338813**	**OK338826**	MH860641
*T. gaditanus*	*Trachyspermi*	DTO 226-B3 = CBS 442.89	Denmark, Lyngby, soil	**OK338793**	**OK338821**	**OK338853**	**OK339615**
*T. gaditanus*	*Trachyspermi*	DTO 228-B8 = CBS 169.81 = IJFM 5146	Spain, Madrid; air; type of *P. gaditanum*	**OK338775**	**OK338802**	**OK338827**	MH861318
*T. gaditanus*	*Trachyspermi*	DTO 333-A5 = CBS 144771	The Netherlands; sputum of cystic fibroses patient	**OK338794**	**OK338822**	**OK338842**	**OK339616**
*T. gaditanus*	*Trachyspermi*	DTO 050-F7 = CBS 444.89	Imported from USA to Denmark; cranberry	**OK338776**	**OK338803**	**OK338828**	**OK339597**
*T. minioluteus*	*Trachyspermi*	DTO 304-C4 = CBS 642.68 = CCRC 31698 = IMI 089377 = LSHB P44 = MUCL 28666 = NRRL 1714	Unknown source and location; neotype of *P. minioluteum*	MN969409	KJ885273	JF417443	JN899346
*T. minnesotensis*	*Trachyspermi*	DTO 423-A7 = CBS 142381 = UTHSC DI16-144 = FMR 14265	USA, Minnesota; human ear; type of *T. minnesotensis*	LT559083	LT795604	LT795605	LT558966
*T. minnesotensis*	*Trachyspermi*	DTO 055-D3	Germany; bread	**OK338795**	**OK338810**	**OK338854**	**OK339617**
*T. minnesotensis*	*Trachyspermi*	DTO 055-D2 = CBS 147315	Germany; wallboard	**OK338796**	**OK338811**	**OK338843**	**OK339618**
*T. minnesotensis*	*Trachyspermi*	DTO 340-G2 = CBS 141838	China; soil	**OK338797**	**OK338823**	**OK338855**	**OK339605**
*T. samsonii*	*Trachyspermi*	DTO 304-C3 = DTO 169-G6 = CBS 137.84 = CECT 2772 = IMI 282404 = IMI 327872	Spain, Valladolid; apple (*Malus sylvestris*) damaged by insect; type of *P. samsonii*	**OK338798**	**OK338824**	**OK338844**	MH861709
*T. samsonii*	*Trachyspermi*	DTO 392-I9 = CBS 147356	The Netherlands; soil	**OK338777**	**OK338804**	**OK338829**	**OK339598**
*T. samsonii*	*Trachyspermi*	DTO 420-B2 = CBS 147357 = ATHUM 9801	Greece, air in house	**OK338778**	**OK338805**	**OK338830**	**OK339599**
*Talaromyces* sp.	*Trachyspermi*	DTO 111-B3 = CBS 282.59	Unknown location; jute treated with copper-naphthenate	**OK338779**	**OK338806**	**OK338831**	**OK339607**
*Talaromyces* sp.	*Trachyspermi*	DTO 162-E5 = CBS 147336	Germany; lemon solution	**OK338780**	**OK338814**	**OK338846**	**OK339608**
*T. germanicus*	*Trachyspermi*	DTO 055-D1 = CBS 147314	Germany; indoor environment	**OK338799**	**OK338812**	**OK338845**	**OK339619**
*T. chongqingensis*	*Trachyspermi*	DTO 060-C9 = DTO 013-A5 = CBS 270.35 = CBS 147316	USA, Virginia, Castle Rock; *Zea mays*	**OK338781**	**OK338807**	**OK338832**	**OK339609**
*T. chongqingensis*	*Trachyspermi*	CGMCC 3.20482	China; soil, type of *T. chongqingensis*	MZ361343	MZ361350	MZ361357	MZ358001
*T. africanus*	*Trachyspermi*	DTO 179-C5 = KAS 3859 = CBS 147340	South Africa; house dust	**OK338782**	**OK338808**	**OK338833**	**OK339610**
*T. trachyspermus*	*Trachyspermi*	DTO 149-H6 = CBS 373.48 = ATCC 10497 = IFO 31757 = IMI 040043 = NRRL 1028 = QM 7682	USA; unknown substrate; type of *T. trachyspermus*	**OK338800**	KJ885281	JF417432	MH856401
*T. udagawae*	*Trachyspermi*	DTO 302-A8 = CBS 579.72 = FRR 1727 = IFO 8808 = IMI 197482 = NHL 6089	Japan, Misugimura; soil; type of *T. udagawae*	**OK338783**	KX961260	MN969148	JN899350
*T. flavus*	*Talaromyces*	DTO 310-A2 = CBS 310.38	New Zealand; unknown substrate; type of *T. flavus*	JX494302	KF741949	JF417426	MH867464
*T. sayulitensis*	*Talaromyces*	DTO 304-E4 = ATCC 4713 = ATCC 52244 = FRR 1064 = IBT 4302 = MUCL 29225 = NRRL 1064 = NRRL 1142 = UPSC 3133	USA, Virginia, Castle Rock; *Zea mays*; type of *P. purpurogenum* *var. rubrisclerotium*	**OK338784**	**OK338809**	**OK338834**	KM066172
*T. sayulitensis*	*Talaromyces*	DTO 245-H1 = CBS 138204	Mexico, Sayulita; house dust; type of *T. sayulitensis*	**OK338801**	**OK338825**	MN969146	**OK339606**

^1^ Sequences generated in this study are indicated in bold.

**Table 2:** Overview of microscopic features and colony characters on various media.

Species Name	Ascomata	Growth Rate (mm)			CYA	CYA	MEA	Conidia		
		CYA 30	CYA	MEA	Reverse	Soluble Pigment	Colony Texture	Ornamentation	Shape	Size (μm)
*T. africanus*	absent	18–20	21–23	26–28	reddish brown (8E6)	absent	velvety to weakly floccose	finely roughened	subglobose	2.5–3.5 × 2–2.5
*T. calidominioluteus*	absent	(20–)25–29	20–30	(22–)25–30	concentric rings of light brown (6D8), then brownish orange (6C6), dark brown (6F8), edge orange (6B6–7)	absent	velvety to weakly floccose or cottony	smooth	broadly ellipsoidal	2.5–4 × 2–2.5
*T. chongqingensis*	absent	6–8	17–18	28–29	violet brown (10D8), edge red (10B8)	red to dark red (10B8–10C8)	densely cottony	smooth	ovoidal	2.5–3.5 × 1.5–2.5
*T. gaditanus*	absent	12–16	19–25	25–28	brown (6D8) at the center, orange (5B8–6B8) elsewhere	absent	floccose	smooth	fusiform	2.5–4 × 2–2.5(–3)
*T. germanicus*	absent	6–7	20–22	23–25	violet brown (10F7) centrally, brownish red (10D7) elsewhere	cherry red (10B8)	velvety	smooth	narrow ellipsoidal to slightly fusiform	2.5–3.5(–4) × 1.5–2.5
*T. minioluteus*	absent	9–14	17–19	21–26	light brown (6D8) in center, dark brown (6F8) in a ring under dense sporulation, edge sometimes orange (6B8)	light brow (6D8), brownish orange (7C6–8)	funiculose	smooth	broadly fusiform or ellipsoidal	2.5–3.5 × 1.5–2.5
*T. minnesotensis*	absent	20–24	26–27	28–30	dark brown (8F7) at the center, reddish orange (7A7)	absent	granulose	smooth	ellipsoidal	2.5–3.5(–4.5) × 1.5–2.5
*T. samsonii*	absent	11–16	15–21	22–26	violet brown to deep red (10E8–F8, 7B–C8)	vivid red to red (10A8–B8)	floccose	smooth	ellipsoidal to fusiform	2.5–4(–5) × 1.5–3
*T. udagawae*	present	5–8	5–9	15–19	brownish orange (5C5)	absent	floccose	smooth	subglobose to ellipsoidal	3–4 × 2–3

## Data Availability

All sequences generated in this study have been submitted in the GenBank.

## Supplementary Materials

The following are available online at https://www.mdpi.com/article/10.3390/jof7110993/s1, Figure S1: Maximum Likelihood (ML) tree inferred based on the ITS sequence dataset with GenBank sequences (indicated in red). The BI posterior probabilities and ML bootstrap percentages are presented at the nodes. The fully supported branches are thickened and the BS/PP values are indicated with asterisk. Values less than 70% bs (ML) or less than 0.95 pp (Bayesian analysis) are indicated with a hyphen or not shown. The phylogram is rooted with *T. flavus* (CBS 310.38^T^) and *T. sayulitensis* (CBS 138204). ^T^ = ex-type, Table S1: Re-identification of *T. minioluteus* sequences present in GenBank.
