# Supplementary material for: Re-Evaluation of the Taxonomy of Talaromyces minioluteus
Source: J Fungi (Basel). 2021 Nov 20;7(11):993. doi: 10.3390/jof7110993 (PMC8619165; doi:10.3390/jof7110993)

Suppl. Table S1. Re-identification of *T. minioluteus* sequences present in GenBank.

GenBank no.	Old name	Current name	Strain / Isolate	Location/substrate
FJ004304	*T. minioluteus*	*T. minioluteus*	ATHUM 5049	Greece, Athens; air, dairy industry
KF471124	*T. minioluteus*	*T. calidominioluteus*	PILE_14-5	Thailand; marine isolate
KP764911	*T. minioluteus*	*Talaromyces* sp.	PmKSA13-01	Saudi Arabia; air, Al-Masjid an-Nabawi Mosque
KR909188	*T. minioluteus*	*T. gaditanus*	PEN3	USA; grapevine wood
KY379613	*T. minioluteus*	*Talaromyces* sp.	SNT2-23	China; endophytic, in parasitic plant *Cynomorium songaricum* and its host *Nitraria tangutorum*
LC195241	*T. minioluteus*	*T. gaditanus*	T4906-7-3	Japan; painting, stone chamber of the Takamatsuzuka Tumulus
LC195244	*T. minioluteus*	*T. gaditanus*	T7521-8F-1	Japan; plastic cover, stone chamber of the Takamatsuzuka Tumulus
LC195245	*T. minioluteus*	*T. gaditanus*	T7521-8H-1	Japan; plastic cover, stone chamber of the Takamatsuzuka Tumulus
LC195246	*T. minioluteus*	*T. gaditanus*	T7530-16-2	Japan; plaster pieces, stone chamber of the Takamatsuzuka Tumulus
LC195247	*T. minioluteus*	*T. minioluteus*	T7615-5-2	Japan; wall, stone chamber of the Takamatsuzuka Tumulus
LC195249	*T. minioluteus*	*T. minioluteus*	K8626-8	Japan; soil in burial mound of the Kitora Tumulus
LC195250	*T. minioluteus*	*T. minioluteus*	K101008-7-6	Japan; clay soil, Kitora Tumulus
MK841457	*T. minioluteus*	*Talaromyces* sp.	voucher_NWUSeq45	South Africa; groundnut
MN311448	*T. minioluteus*	*T. calidominioluteus*	DnjP/2	Serbia; fruit, *Cydonia oblonga*
MN311450	*T. minioluteus*	*T. calidominioluteus*	ParP/2	Serbia; fruit, *Solanum lycopersicum*
MN311451	*T. minioluteus*	*T. calidominioluteus*	PP/14	Serbia; fruit, *Citrus sinensis*
MT074701	*T. minioluteus*	*T. gaditanus*	3107	Malaysia; soil
MT079313	*T. minioluteus*	*Talaromyces* sp.	NWU45	South Africa; peanuts
MT441589	*T. minioluteus*	*T. minnesotensis*	2nce	Chile; root tissues, *Aristolochia chilensis*
MT738204	*T. minioluteus*	*T. minnesotensis*	CT1-013A	Canada; plant material
MT872096	*T. minioluteus*	*T. calidominioluteus*	KrP/7	Serbia; fruit, *Pyrus communis* (common pear)
MT940692	*T. minioluteus*	*T. calidominioluteus*	PUMCH_Q032	China; tracheal aspirates, *Homo sapiens*
MW405459	*T. minioluteus*	*Talaromyces* sp.	6FP9	Iran; periphytic biofilm and rhizosphere of rice
MW540483	*T. minioluteus*	*T. gaditanus*	4.25	Croatia; *Quercus robur* acorn
MW793373	*T. minioluteus*	*Talaromyces* sp.	UASWS2526_PD1-12	Algeria; endophytic, *Pancratium maritimum* on sand dune
MW826207	*T. minioluteus*	*T. samsonii*	240315GAR3C2	Spain; air, La Garma Cave

Suppl. Figure S1: ITS

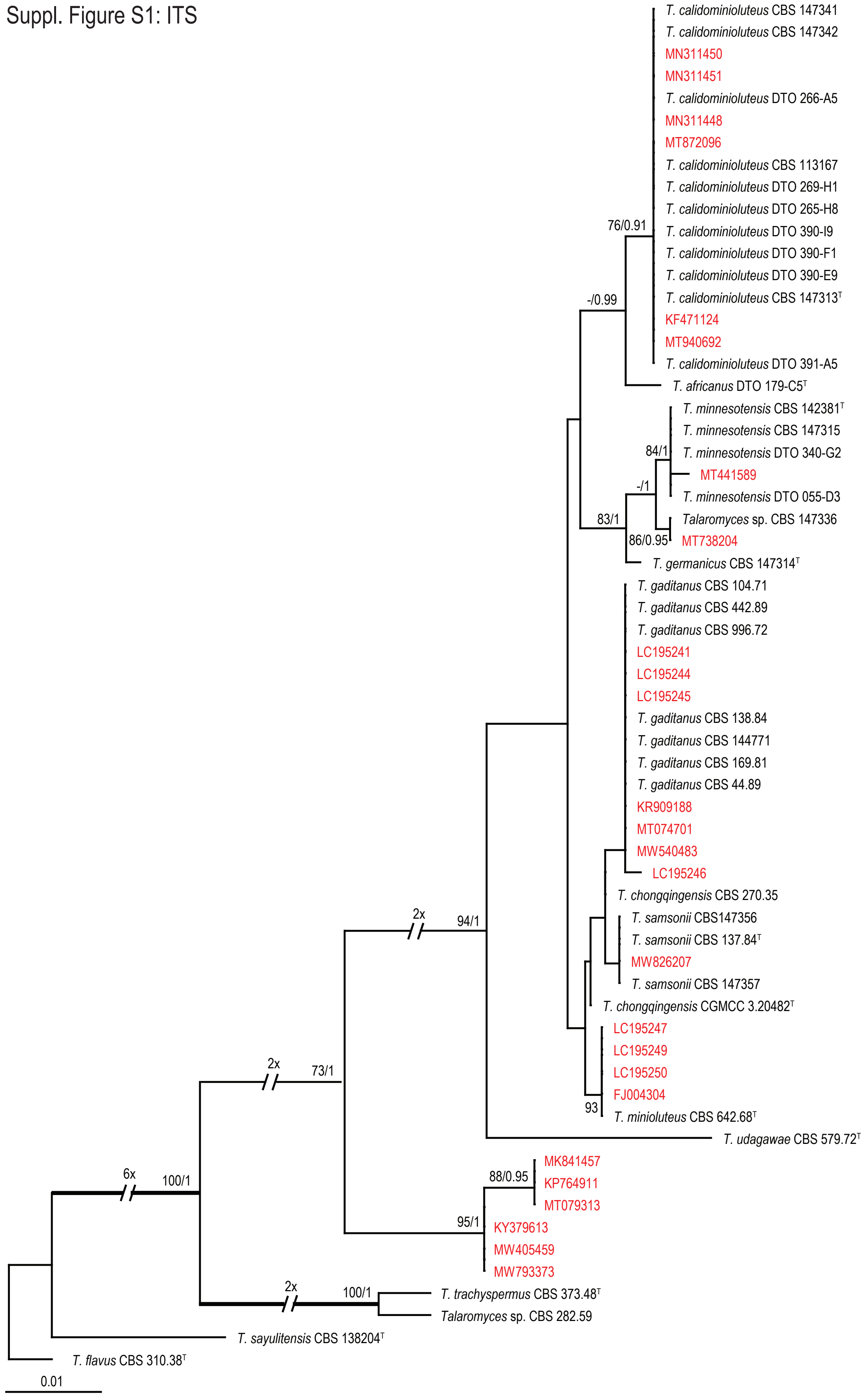

Supplement: Supplementary file 1 [file jof-07-00993-s001.zip › jof-1452926-supplementary.pdf]
